# Identifying essential long non-coding RNAs in cancer using CRISPRi-based dropout screens

**DOI:** 10.1016/j.xpro.2023.102588

**Published:** 2023-09-28

**Authors:** Oscar Bril, Laura J. Schwarzmueller, Leandro F. Moreno, Louis Vermeulen, Nicolas Léveillé

**Affiliations:** 1 Center for Experimental and Molecular Medicine, Cancer Center Amsterdam and Amsterdam Gastroenterology Endocrinology Metabolism, Amsterdam UMC, University of Amsterdam, Meibergdreef 9, Amsterdam 1105AZ, The Netherlands; 2 Oncode Institute, Meibergdreef 9, Amsterdam 1105AZ, The Netherlands

**Keywords:** Bioinformatics, Cell Biology, Genomics, Molecular Biology, CRISPR

## Abstract

Long non-coding RNAs (lncRNAs) are emerging as key regulators in the initiation, growth, and progression of cancer. High-throughput CRISPR-based techniques systematically assess the function of genes or regulatory elements present in the human genome. Here, we present a protocol for identifying essential lncRNAs in cancer using CRISPRi-based dropout screens. We describe steps to select target sites, design guide RNAs, and generate CRISPRi cell lines. We then detail the execution and analysis of CRISPRi-based dropout screens.

## Highlights

• Comprehensive detection of expressed lncRNAs using GRO-Seq
• Definition of target regions and automated gRNA design
• Description of a CRISPRi-based dropout screen to uncover essential lncRNAs
• Analysis of CRISPRi screen data

## Abstract (editor highlights)

Publisher’s note: Undertaking any experimental protocol requires adherence to local institutional guidelines for laboratory safety and ethics.

## Graphical abstract

**Figure:**
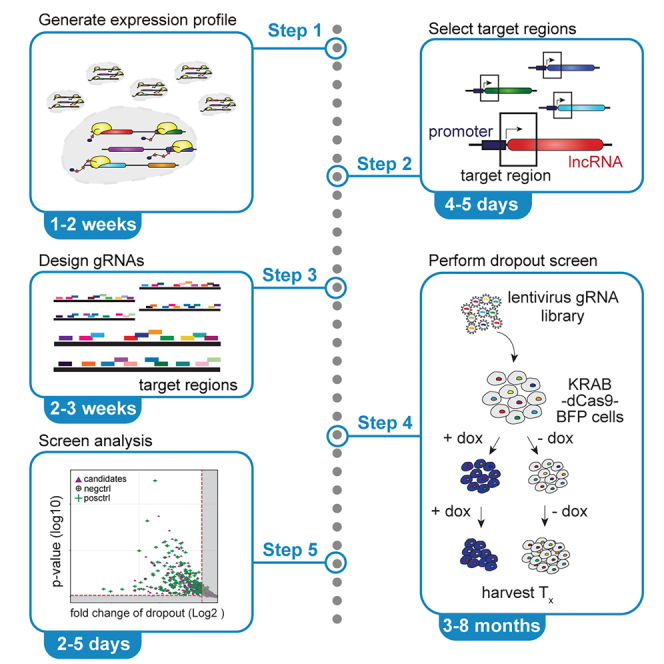

## Before you begin

**Figure 1:**
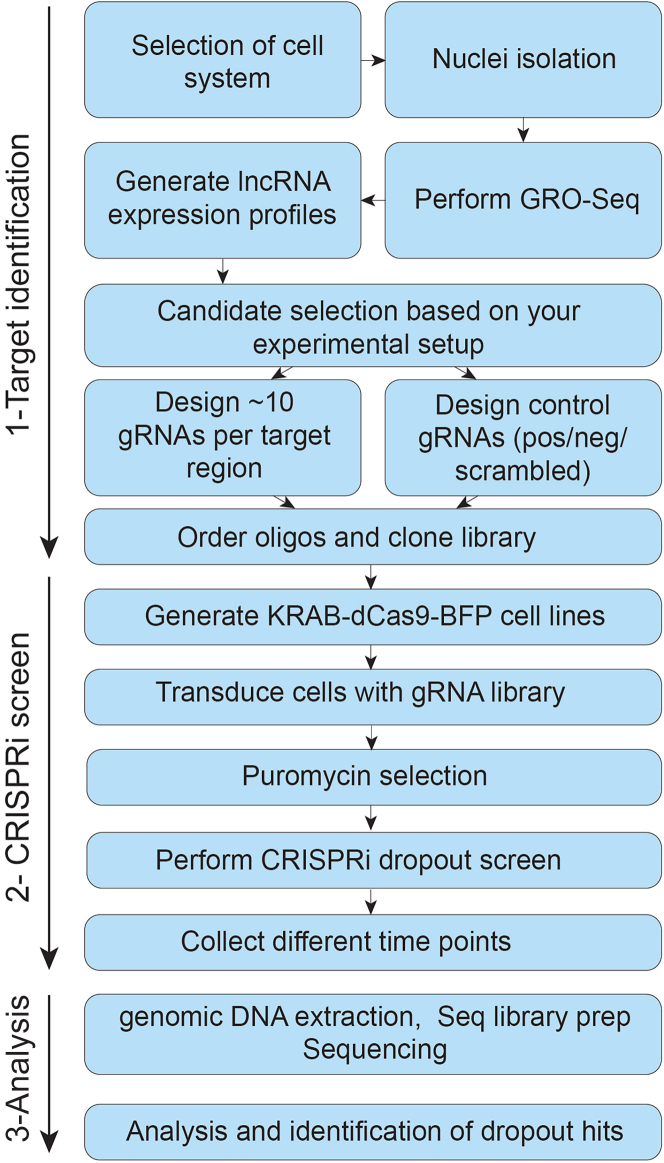
Workflow to assess the function of expressed/selected lncRNAs with CRISPRi dropout screens The schematic illustrates essential steps for each section of the protocol, including 1-Target identification, 2-CRISPRi screen and 3-Screen analysis.

This protocol describes the nascent RNA profiling of *in vitro*-cultured cell lines with subsequent functional assessment of selected transcripts using a CRISPRi-based screening approach. While we established this procedure using a panel of colorectal cancer (CRC) cell lines (e.g., Colo320HSR, LS180, HT55, LS411N), most *in vitro*-cultured cellular models can be used. A schematic overview of the workflow is shown in Figure 1.

### Target identification

This section describes the capture of transcriptional changes associated to specific biological contexts of interest by performing Global run-on sequencing (GRO-Seq). We generally apply GRO-seq to monitor the transcriptional regulation of lncRNAs in response to the disruption of specific transcription factors in a cellular context of interest, and subsequently assess the function of identified deregulated transcripts by CRISPRi-based dropout screens. While this method can be used to identify new essential molecules related to specific transcriptional programs, it can also be employed to uncover key transcriptional changes following specific treatments (e.g., chemotherapy) or to characterize specific cellular states (e.g., chemo-sensitive vs. chemo-resistant cells). Alternatively, GRO-seq can be used to identify all expressed lncRNAs in a cellular context of interest.

### CRISPRi screen

Here, we describe the design of gRNAs to target the transcription start sites (TSSs) of selected lncRNAs, using the GRO-Seq data generated in the first part of this protocol. In addition, control gRNAs, which are crucial for downstream analyses, must be included. Among the various controls, we systematically include gRNAs targeting essential (coding) genes (positive controls) that have been previously described and that are expressed in the selected cell system. We also include non-targeting scrambled gRNAs (negative control group 1) that do not align to any genomic sequence in the used model system. A group of gRNAs targeting non-essential^1^ or non-expressed genes (negative control group 2) can also be added. Control gRNA sequences are provided in Table S1.

Prior to performing a CRISPRi-based dropout screen, plasmid vectors expressing both KRAB-dCas9 and the gRNAs must be selected. Special attention should be given to ensure complementary selection markers (fluorescent proteins and/or resistance genes). In addition, if a doxycycline (dox) inducible system is preferred, the amount of dox required to adequately control the desired expression in your cell system of interest should be thoroughly tested.

Dropout screens will generally be analyzed over several time points (e.g., day 0, 10 and 20). The ideal harvesting time points may vary between different cell models, which are likely to present distinct doubling times and sensitivities. Therefore, we recommend harvesting multiple time points over a period of 2–3 weeks to ensure the capture of dropout hits.

### Screen analysis

This section details the procedure to analyze each CRISPRi screen and steps to produce a list of potential dropout candidates. Utilization of specific packages such as MAGeCK and DESeq2, as well as arbitrary cutoffs and filters are discussed.

## Key resources table

**Table:** 

REAGENT or RESOURCE	SOURCE	IDENTIFIER
**Antibodies**		

BrdU Antibody (IIB5) AC	Santa Cruz	Cat#sc-32323 AC

**Chemicals, peptides, and recombinant proteins**		

Doxycycline	Sigma-Aldrich	Cat#D9891
UltraPure™ DNase/RNase-Free Distilled Water	Thermo Fisher Scientific	Cat#10977-049
5-Bromouridine 5′-triphosphate sodium salt	Sigma-Aldrich	Cat# B7166
ATP	Thermo Fisher scientific	Cat#AM8110G
CTP	Thermo Fisher scientific	Cat#AM8120G
GTP	Thermo Fisher scientific	Cat#AM8130G
SUPERaseIN	Thermo Fisher scientific	Cat#AM2696
Sarkosyl	Sigma-Aldrich, Merck	Cat#L7414
KCl	Thermo Fisher scientific	Cat#AM9640G
MgCl_2_	Thermo Fisher scientific	Cat#AM9530G
CaCl_2_	Sigma-Aldrich, Merck	Cat#21115-100ML
NaCl	Thermo Fisher scientific	Cat#AM9760G
UltraPure™ 1 M Tris-HCI, pH 7.5	Thermo Fisher Scientific	Cat#15567027
UltraPure™ 1 M Tris-HCI, pH 8.0	Thermo Fisher Scientific	Cat#15568025
Tris-HCl, pH 8.3	N/A	N/A
Glycerol	Sigma-Aldrich	Cat#G5516
EDTA	Thermo Fisher Scientific	Cat# AM9261
Tris-EDTA, pH 7.4	N/A	N/A
SDS solution, 10% sodium dodecyl sulfate solution	Thermo Fisher Scientific	Cat#10552785
IGEPAL	Thermo Fisher Scientific	Cat#AM9261
DTT	Thermo Fisher Scientific	Cat#D-1532
Trizol LS	Thermo Fisher Scientific	Cat#10296010
SSPE	Thermo Fisher Scientific	Cat#AM9767
Polyvinylpyrrolidone (PVP)	Sigma-Aldrich	Cat# PVP360
Acid phenol-chloroform ph4.5 (with IAA)	Thermo Fisher scientific	Cat#AM9720
GlycoBlue™ Coprecipitant	Thermo Fisher scientific	Cat#AM9516
ultrapure BSA	Thermo Fisher scientific	Cat#AM2616
Phosphate-buffered saline (PBS)	Fresenius Kabi	N/A
Trypsin-EDTA 0.5%	Gibco Life science	Cat#15400-054
Hexadimethrine bromide (Polybrene)	Sigma-Aldrich	Cat# 107689
Chloroform	N/A	N/A
Tween20	Sigma-Aldrich	N/A
Ethanol	N/A	N/A
Isopropanol	N/A	N/A
Lipofectamine 2000	Thermo Fisher Scientific	Cat#11668-019

**Critical commercial assays**		

TruSeq Stranded mRNA Library Prep Kit	Illumina	Cat#20020596 (not available anymore) Alternative: NEBNext Ultra II Directional RNA Library Kit Cat#E7760
NEBNext® Library Quant Kit for Illumina	New England Biolabs	Cat#E7630L
Genomic DNA Purification Kit	Thermo Fisher Scientific	Cat#K0512
NucleoSpin Gel & PCR Clean-up	Bioké	Cat#740609

**Deposited data**		

Human reference genome NCBI build 37, GRCh38/hg38	Genome Reference Consortium	http://www.ncbi.nlm.nih.gov/projects/genome/assembly/grc/human/

**Experimental models: Cell lines**		

Human: HEK293		
Human: LS180		
Human: HT55		
Human: LS411N		
Human: COLO320HSR		

**Oligonucleotides**		

gRNAs for CRISPRi screen, see Table	Twist Bioscience	Customized
PCR1 primer pDecko-puro-mCherry forward	GAGGGCCTATTTCCCATGATTC	
PCR1 primer pDecko-puro-mCherry reverse	TAAAATTGTGGATGAATACTGCCAT	

**Recombinant DNA**		

TRE-KRAB-dCas9-IRES-BFP	Addgene	#85449
pDECKO-mCherry	Addgene	#78534
pHR-EF1Alpha-Tet-on 3G	Addgene	#118592

**Software and algorithms**		

FlowJo Software	BD Biosciences	https://www.flowjo.com/solutions/flowjo/downloads
Windows Subsystem for Linux (WSL)	Microsoft	https://learn.microsoft.com/en-us/windows/wsl/install
Python 3.8 or above	Python software foundation	https://www.python.org/
Ubuntu 20.04 or similar UNIX system	Canonical Ltd.	https://ubuntu.com/download/desktop
R version 4.2 or above	R foundation	https://cran.r-project.org/
R studio	Posit Software, PBC formerly RStudio, PBC	https://posit.co/downloads
FastQC	Babraham Institute Bioinformatics	https://www.bioinformatics.babraham.ac.uk/projects/fastqc/.
STAR	Dobin et al.^2^	https://github.com/alexdobin/STAR
NRSA	Wang et al.^3^	https://zenodo.org/badge/latestdoi/680108742
CCTop	Stemmer et al.^4^	https://bitbucket.org/juanlmateo/cctop_standalone/src/master/
BLAT	UC Santa Cruz	https://users.soe.ucsc.edu/∼kent/src/
Trimmomatic v.0.39	Bolger et al.^5^	http://www.usadellab.org/cms/?page=trimmomatic
MAGeCK	Li et al.^6^	https://bitbucket.org/liulab/mageck/src/master/
DESeq2 R package	Love et al.^7^	https://bioconductor.org/packages/release/bioc/html/DESeq2.html
EnhancedVolcano R package	Blighe et al.^8^	https://bioconductor.org/packages/release/bioc/html/EnhancedVolcano.html

**Other**		

Culture flask 50 mL 25 cm^2^	Greiner Bio-One	Cat#690175
Culture flask 250 mL 75 cm^2^	Greiner Bio-One	Cat#658175
CELLSTAR 6 well culture plates	Greiner Bio-One	Cat#657160
CELLSTAR Cell Culture Dish with Lid, 100 mm diameter	Greiner Bio-One	Cat#664160
CELLSTAR Cell Culture Dish with Lid, 145 mm diameter	Greiner Bio-One	Cat#639160
15 mL tubes	Greiner Bio-One	Cat#188271
50 mL tubes	Greiner Bio-One	Cat#227261
5 mL FACS tubes	Corning	Cat#352008
P1250 Long Reach Filter Tips	Westburg	Cat#WS5074
P200 Long Reach Filter Tips	Westburg	Cat#WS5040
P20 Long Reach Filter Tips	Westburg	Cat#WS5020
P10 Long Reach Filter Tips	Westburg	Cat#WS5010
1.5 mL tubes, DNAse/RNAse free	Greiner Bio-One	Cat#616201
Nonstick, RNase-free microfuge tubes, 1.5 mL	Ambion, Thermo Fisher Scientific	Cat#AM12450

## Materials and equipment

**Table:** 

Equipment	Source	Identifier
Panasonic CO2 incubator	Panasonic Healthcare corporation	MCO-170AICUVH-PE
Hettich ROTINA 420R centrifuge	Hettich zentrifugen	Z723754
SH800 Cell Sorter	Sony Biotechnology	N/A
Counting chamber	N/A	N/A
Light microscope	N/A	N/A
Thermomixer	N/A	N/A
Stuart rotator SB3	Stuart	N/A
Bioanalyzer 2100	Agilent	N/A
Hardware	Comment	Benefit
≥32 GB of RAM	To load the human genome in its entirety while mapping	Improves performance
≥8 cores	To use multithreading	Improves performance drastically

## Step-by-step method details

### Target identification

#### Selection of cell system and nuclei isolation

**Figure 2:**
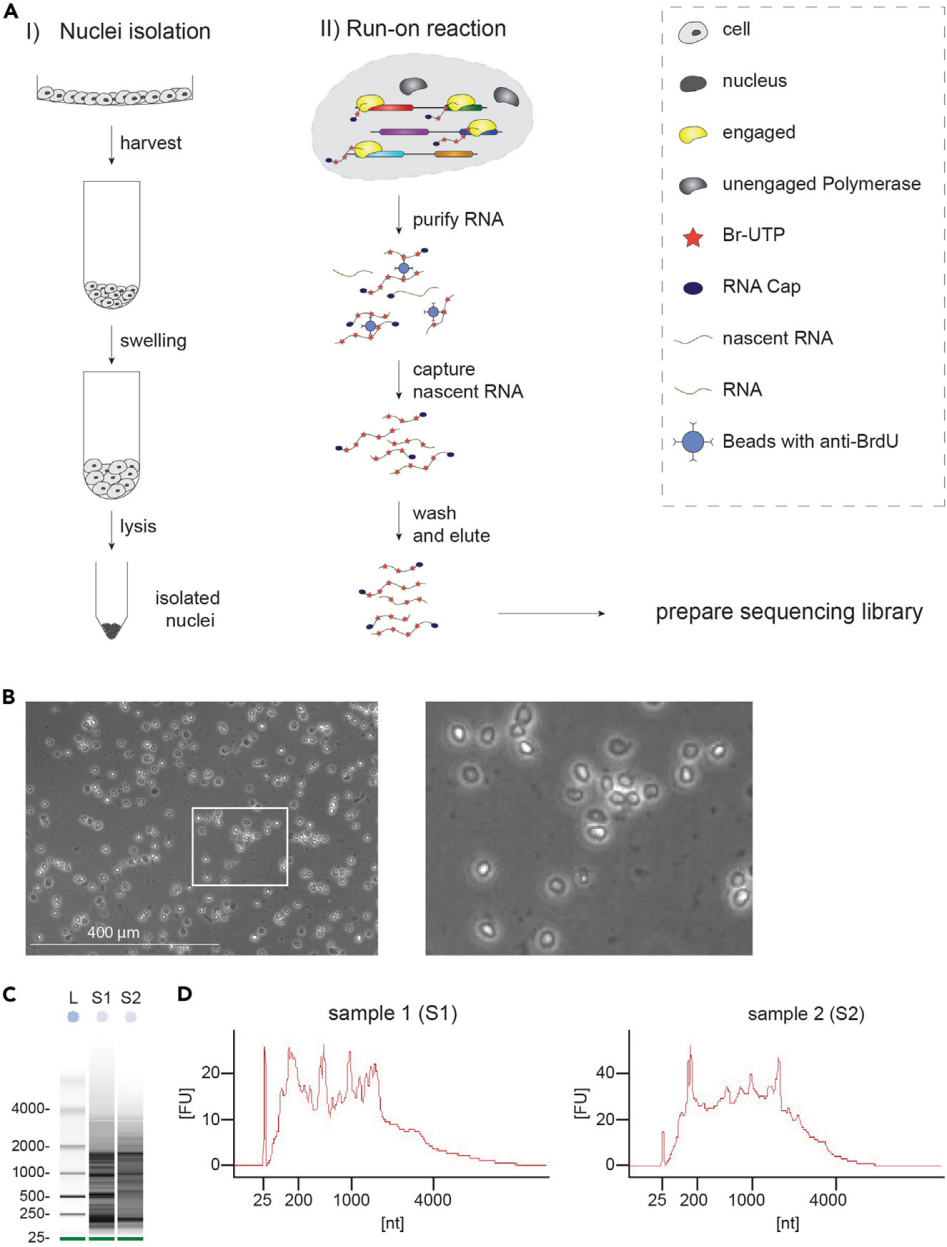
Global run-on sequencing (A) Scheme highlighting key steps required to perform global run-on sequencing. (B) Example of nuclei obtained from the CRC cell line HCT116, following the procedure illustrated in A1. (C) Analysis of two purified run-on reactions (S1 and S2) using a Bioanalyzer RNA 6000 Pico Chip. The size distribution is indicated by a ladder (L). (D) Electropherograms of samples presented in B. Fragment sizes (nucleotides, nt) are plotted against arbitrary fluorescence units (FU).

An adequate cell system or cell lines of interest should be initially selected. Any cell line that can be expanded *in vitro* is in principle suited for this protocol. Our laboratory mainly focuses on colorectal cancer and we, therefore, established this procedure using a panel of CRC cell lines (e.g., Colo320HSR, LS180, HT55 and LS411N). Each cell line is then profiled using GRO-seq. To capture nascent transcripts, millions of cell nuclei are isolated and transcription is reinitiated in presence of an analog of UTP (Br-UTP). Nascent transcripts with incorporated Br-UTP can be captured with an agarose-conjugated anti-BrdU antibody (Santa Cruz, IIB5). This section describes how to isolate cell nuclei, perform and analyze GRO-seq. Steps related to the GRO-Seq protocol are illustrated in Figure 2A.

**CRITICAL:** RNA is sensitive to temperature and enzymatic digestion by RNases, which are normally present on skin, surfaces, etc. Keep all surfaces and equipment clean while performing this protocol. We also recommend to wear gloves and to keep samples on ice whenever indicated in the protocol. Only use RNase-free materials and chemicals.

***Note:*** Here we describe the procedure from the day cells are harvested for nuclei isolation. We do not consider seeding density, expansion time and possible treatments. However, if you have different treatments or conditions in your experimental setup, these factors should be carefully considered in the overall planning. For cancer cell lines, we generally harvest 20 × 10^6^ cells per replicate and combine two run-on reactions (each with 5 × 10^6^ nuclei) in order to obtain sufficient material for the preparation of a sequencing library. However, if the transcriptional output of your cells of interest is low, you may have to increase the number of pooled run-on reactions per replicate.

#### Day 1

1. Prepare the swelling, lysis, and freezing buffers and pre-cool them on ice.
2. Cool down a centrifuge to 4°C and pre-warm the trypsin.
3. Trypsinize the cells until they become rounded and floating.
  a. Neutralize the trypsin with 2 volumes of 10% FBS-supplemented medium and transfer each sample to a 50 mL tube. Make sure that cells are properly singularized.
  b. Centrifuge samples for 5 min at 300 × *g*, and discard the supernatants.
  c. From here on, keep the pellets on ice unless indicated otherwise.
4. Resuspend each pellet in 30 mL of swelling buffer.
  a. Incubate samples 10 min on ice with occasional inverting of the tubes.
  b. Centrifuge samples for 5 min at 500 × *g* (4°C).
  c. Carefully remove the supernatant without disturbing the pellet.
5. Resuspend each pellet in 10 mL of lysis buffer and transfer the suspension into a 15 mL tube.
  a. Invert the tube 30 times and spin down for 5 min at 500 × *g* (4°C).
  b. Remove the supernatant.
6. Repeat step 5.

***Note:*** If a large number of cells (> 5 × 10^7^) is collected per sample, you may split them in several tubes to obtain a better swelling and lysis reactions. You can visually inspect your sample under the microscope (after step 6), and confirm the presence of clean nuclei (see Figure 2B). If not, you can repeat step 5, before resuspending the sample in freezing buffer.

7. Resuspend the pellet in 1 mL of freezing buffer and transfer the suspension to a 1.5 mL tube.
  a. Count nuclei using trypan blue.
  b. Spin down samples for 5 min at 500 × *g* (4°C).
  c. Resuspend the nuclei in freezing buffer to reach a concentration of 5 × 10^6^ nuclei per 100 μL of buffer.
  d. Aliquot 100 μL into 1.5 mL tubes and keep them on ice.

**Pause point:** Here you can STOP and freeze nuclei aliquots at −80°C OR proceed to the run-on reactions.

***Note:*** For each replicate, we recommend to perform two run-on reactions (each with 5 × 10^6^ nuclei). If your harvest did not yield enough nuclei for two run-on reactions, you can repeat the previous steps or control the efficacy of your nuclei isolation by taking along cell lines with high nuclei isolation yield such as HCT116 and HEK293.

### Run-on reactions

**Timing: 1 h**

Here we describe the use of global run-on sequencing to identify expressed or regulated lncRNAs in a cell line of interest. Compared to conventional RNA-Seq, GRO-Seq captures nascent transcripts, which improves the detection of less stable and lowly transcribed RNAs (including non-polyA RNAs).^9^^,^^10^ In addition, this method allows the precise mapping of transcription start sites (TSSs). This feature is particularly important since current CRISPRi tools achieve best transcriptional regulation when gRNAs are targeted to these regions.

8. Preheat a thermo-mixer to 30°C.
9. Prepare the reaction buffer according to Table 2 and preheat to 30°C.

**Table 1:** Buffers for nuclei isolation

Buffer	Reagent	Final concentration
Swelling Buffer	Tris-HCL, pH 7.5	10 mM
	MgCl_2_	2 mM
	CaCl_2_	3 mM
	RNase-free H_2_O	N/A
Lysis Buffer	Tris-HCL, pH 7.5	10 mM
	MgCl_2_	2 mM
	CaCl_2_	3 mM
	IGEPAL	0.5%
	Glycerol	10%
	RNase-free H_2_O	N/A
Freezing Buffer	Tris, pH 8.3	50 mM
	Glycerol	40%
	MgCl_2_	5 mM
	EDTA	0.1 mM
	RNase-free H_2_O	N/A

Composition of buffers for nuclei isolation.

**Table 2:** Buffer to perform run-on reactions

Reagent	Final concentration
Tris, pH 8.0	10 mM
MgCl_2_	5 mM
KCl	300 mM
Sarkosyl	1%
DTT	1 mM
ATP, GTP, Br-UTP	500 μM
CTP	2 μM
SUPERaseIN	20 U
RNase-free H_2_O	N/A

Composition of buffers for run-on reactions.
10. If you have stored the nuclei at −80°C, thaw them on ice.
11. Add 100 μL of pre-warmed reaction buffer (1× volume) to each reaction tube with 100 μL of nuclei and keep tubes at 20°C.
  a. Cut off the tip of a 200 μL pipette tip (the mixture is very viscous) and pipette up and down each reaction mixture. Change the tip between each sample to avoid contamination. Pipette carefully to avoid air bubbles.
  b. Incubate samples for 5 min at 30°C.
  c. Pipette the reaction mixtures up and down (using cutoff tips) after 2–3 min of incubation, while keeping the samples at 30°C. Pipette gently and avoid making air bubbles.
12. Add 600 μL (3× volume) of Trizol LS to each reaction tube and mix samples by vortexing.
  a. Incubate samples for 5 min at 20°C.

**Pause point:** You can STOP here and store samples at −80°C, according to the manufacturer’s recommendations OR proceed with the RNA purification.

### RNA purification and capture of nascent transcripts

**Timing: 7–12 h over 3 days**

#### Day 1

**Timing: 2–3 h**

Here we detail the methods used to purify nascent RNAs and prepare sequencing libraries.

13. Pre-cool a tabletop centrifuge to 4°C.
14. Add 160 μL of chloroform to each sample.
  a. Vortex samples for 15 s and incubate for 3 min at 20°C.
  b. Centrifuge samples for 15 min at 12000 × *g* (4°C).
  c. Transfer 400 μL of the aqueous phase (top) into a 1.5 mL collection tube. We recommend transferring 2 × 200 μl (using 200 μl tips) to avoid potential contamination with the interphase.
  d. Discard tubes with the bottom phase.
15. Add 400 μL (1× volume) of acid phenol-chloroform to each sample.
  a. Vortex samples for 30 s.
  b. Centrifuge the tubes for 10 min at 12000 × *g* (4°C).
  c. Transfer 300 μL of the aqueous phase (top) to a new collection tube.
  d. Discard the tubes with the bottom phase.
16. Add 300 μL (1× volume) of chloroform to each sample.
  a. Vortex samples for 30 s.
  b. Centrifuge samples for 10 min at 12000 × *g* (4°C).
  c. Transfer 250 μL of the aqueous phase (top) to a new collection tube.
  d. Combine 2 run-on reactions per sample into one tube (total of 500 μL).
  e. Discard tubes with the bottom phase.
17. Add 2 μL Glyco Blue to each sample.
  a. Vortex vigorously for 10 s.
18. Add 350 μL (0.7 x volume) of isopropanol to each tube.
  a. Vortex vigorously for 15 s.
  b. Incubate samples 14–18 h at −20°C.
19. Prepare buffers as detailed in Table 3.

**Table 3:** Buffers for bead preparation

Buffer	Reagent	Final concentration
Bead blocking buffer	SSPE	0.25%
	EDTA	1 mM
	Tween20	0.05%
	PVP	0.1%
	RNase-free H_2_O	N/A
Bead binding buffer	SSPE	0.25%
	EDTA	1 mM
	NaCl	37.5 mM
	Tween20	0.05%
	Add SUPERaseIN freshly	4 U/mL
	RNase-free H_2_O	N/A

Composition of buffers to prepare anti-BrdU beads.
20. To prepare anti-BrdU beads, transfer 60 μL (for each sample) of the bead solution into a 1.5 mL tube.
  a. Spin down the beads for 30 s at 500 × *g*.
  b. Carefully remove the supernatant.
  c. Add 800 μL of bead blocking buffer per tube.
  d. Spin down the beads for 30 s at 500 × g and remove the supernatant.
21. Repeat step 21.
22. Resuspend the bead pellet in 1 mL of bead blocking buffer.
  a. Add BSA to a final concentration of 1 mg/mL.
23. Incubate the beads on a rotator for 14–18 h (4°C).

#### Day 2

**Timing: 3–5 h**

***Note:*** To minimize the loss of RNA, use low binding 1.5 mL collection tubes for the following steps.

24. Pre-cool a tabletop centrifuge to 4°C.
25. Spin down the BSA-blocked beads for 30 s at 500 × *g*.
  a. Remove the supernatant.
  b. Resuspend each bead pellet in 500 μL of bead binding buffer.
26. Centrifuge RNA samples for 30 min at maximum speed (4°C). A small blue pellet should be visible after the centrifugation step.
  a. Remove the supernatant without disturbing the pellet.
27. Add 800 μL of 75% ethanol to each RNA pellet and vortex the samples for 5 s.
  a. Spin down samples for 5 min at maximum speed (4°C).
  b. Remove the supernatant and let the pellets dry with an open lid on the bench.
  c. Remove residual traces of liquid by carefully flicking the tube.

***Note:*** Make sure that the pellet is completely dry before adding H_2_O.

28. Add 100 μL of H_2_O (supplemented with RNase inhibitor) to each pellet and put the samples on ice.

***Note:*** We use SUPERaseIN RNase inhibitor at a concentration of 20 U in 10 mL of H_2_O. You can use similar products at a comparable concentration.

29. Preheat a thermo-mixer to 70°C.
30. Dissolve RNA pellets for 5 min on ice, and carefully pipette up and down each sample.
31. Incubate samples for 5 min at 70°C.
32. Quickly transfer samples on ice and incubate for 2 min.
  a. Spin down samples for 5–10 s to collect all liquid at the bottom.
33. Transfer each RNA sample (100 μL) to previously prepared tube containing blocked anti-BrdU beads, and mix carefully by pipetting (total volume ∼600 μL).
  a. Incubate samples on a rotor for 2 h at 4°C.
34. During this incubation time, prepare the Low-salt, High-salt, Tris-EDTA-Tween and Elution buffer (without DTT), as specified in Table 4.

**Table 4:** Buffers for RNA purification and capture of nascent transcripts

Buffer	Reagent	Final concentration
Low salt buffer	SSPE	0.25%
	EDTA	1 mM
	Tween20	0.05%
	SUPERaseIN	4 U/mL add before use
	RNase-free H_2_O	N/A
High salt buffer	SSPE	0.25%
	NaCl	137.5 mM
	EDTA	1 mM
	Tween20	0.05%
	SUPERaseIN	4 U/mL add before use
	RNase-free H_2_O	N/A
Tris-EDTA-Tween buffer	Tris-EDTA, pH 7.4	
	Tween20	0.05%
Elution buffer	Tris, pH 7.5	5 mM
	NaCl	300 mM
	EDTA	1 mM
	DTT	20 mM (add before use)
	SDS	0.1%
	RNase-free H_2_O	N/A

Composition of buffers to purify and capture nascent RNA transcripts.
35. Pre-chill the Low-salt, High-salt and Tris-EDTA-Tween buffers on ice.
36. Keep the Elution buffer at 20°C and add DTT just before use.

***Note:*** You can prepare buffers before performing this protocol. However, Tween20-containing buffers should be kept in the dark, and RNase-inhibitors as well as DTT should be freshly added before use.

37. Spin down the beads for 30 s at 500 × *g*.
  a. Carefully remove the supernatant without disturbing the pellet.
38. Start the washing steps by adding 500 μL of Low-salt buffer per sample, and place tubes on a rotator for 5 min at 20°C.
  a. Spin down samples for 30 s at 500 × *g* and remove the supernatant.
  b. Repeat step 38.
39. Add 500 μL of High salt buffer per sample, and place tubes on a rotator for 5 min at 20°C.
  a. Spin down samples for 30 s at 500 × *g* and remove the supernatant.
  b. Repeat step 39.
40. Add 500 μL of Tris-EDTA-Tween buffer per sample, and place tubes on a rotator for 5 min at 20°C.
  a. Spin down samples for 30 s at 500 × *g* and remove the supernatant.
  b. Repeat step 40.
41. Add DTT to the Elution buffer and keep at 20°C.
42. Prepare low binding tubes for the elution step.
43. Add 150 μL of Elution buffer to each sample and incubate on a rotator for 5 min at 20°C.
  a. Spin down samples for 30 s at 500 × *g*.
  b. Transfer each eluate to a low binding tube.
  c. Repeat the elution (step 43) two more times and combine eluates of the same sample (total volume ∼450 μL).
44. Pre-chill a tabletop centrifuge to 4°C.
45. Add 500 μL of acid phenol-chloroform to each sample.
  a. Vortex samples for 30 s.
  b. Centrifuge samples for 5 min at 12000 × *g* (4°C).
  c. Transfer 400 μL of the aqueous phase (top) to a new low binding collection tube.
  d. Discard tubes containing the bottom phase.
46. Add 400 μL (1× volume) of chloroform.
  a. Vortex samples for 30 s.
  b. Centrifuge samples for 5 min at 12000 × *g* (4°C).
  c. Transfer 400 μL of the aqueous phase (top) to a new low binding collection tube.
  d. Discard tubes containing the bottom phase.
47. Add 1 μL of Glyco Blue to each sample.
  a. Vortex vigorously for 10 s.

***Note:*** Here we use less Glycogen as compared to the previous steps to avoid inhibition of the reverse transcriptase.

48. Add 1100 μL (2–3× volume) of 100% ethanol per sample.
  a. Vortex vigorously for 15 s.
  b. Incubate samples for 14–18 h at −20°C.

#### Day 3

**Timing: 2–4 h**

49. Pre-chill a tabletop centrifuge to 4°C.
50. Spin samples for 30 min at maximum speed (4°C).
  a. Remove the supernatant.
  b. Dry pellets and remove residual liquid around the pellet.

***Note:*** You should see a small, light blue pellet. If there is no pellet visible, the yield is probably too low to continue.

51. Carefully add 6 μL of H_2_O (supplemented with 1 μL of SUPERaseIN per 10 mL) to each pellet and incubate tubes for 2 min on ice.
  a. Vortex samples and spin down to collect the liquid at the bottom.
  b. Keep samples on ice.

***Note:*** It is recommended to run a small amount of each RNA sample (0.8 μL) on a Bioanalyzer instrument (Agilent) to visually inspect the quality of the run-on, before proceeding to the sequencing library preparation. An example of two run-on reactions analyzed with a Bioanalyzer (Eukaryote Total RNA, RNA 6000 Pico Chip) are shown in Figures 2C and 2D. To avoid RNA degradation, we recommend proceeding immediately to the 1^st^ strand synthesis step.

52. Proceed with the preparation of the sequencing library according to the manufacturer’s manual.

***Note:*** We generated the libraries for sequencing using the TruSeq Stranded mRNA Library Prep Kit (Illumina). Using a stranded sequencing kit is essential to determine strand-specific transcriptional activities. Moreover, we quantified libraries with the NEBNext Library Quant Kit (New England Biolabs). Barcoded samples were pooled equimolarly and sequenced on the Illumina HiSeq4000 platform (single-end 50 bp reads).

### Analysis of GRO-Seq and generation of output matrices

**Timing: 2–3 h per sample**

In this section, we process the GRO-Seq data to identify expressed or differentially regulated genes. Troubleshooting 2 & Troubleshooting 3.

**CRITICAL:** The analysis was executed and tested on an Ubuntu (version 20.04) virtual environment via Windows Subsystem for Linux (WSL, version 1).

**Before you start:** Download the output files from the GRO-Seq and install python. Using a package manager like anaconda with useful pre-installed packages (including jupyter notebook) is highly recommended. Create a new directory called “CRISPRi” for all the data ($DIR from now on). Download the “Cleanup and guide picking.html”, “Ordered csv.html”, and “Merge files.html” files (Supplementary files) and place them in the CRISPRi folder.

53. In order to identify low quality regions in the generated reads, install and run the tool FastQC.

***Note:*** In general, reads classified with a green flag are high-quality reads and will be used for the GRO-seq analysis. In some cases, the reads classified with yellow flags require further attention. In particular, rRNA contamination and other quality issues should be analyzed, and if the quality of the reads is too low, re-sequencing is necessary.

54. To remove low-quality sequences identified in the previous step, download and install the tool Trimmomatic.^5^

***Note:*** The command line code used in this step will depend on the issue identified by the FASTQC tool. Therefore, we recommend to carefully read the documentation of Trimmomatic and correct the reads accordingly.

55. To map high-quality reads to the human reference genome with the STAR aligner tool,^2^ download necessary files to generate the reference genome:

$ wgethttps://ftp.ebi.ac.uk/pub/databases/gencode/Gencode_human/release_XX/GRCh38.primary_assembly.genome.fa.gz.

$ wgethttps://ftp.ebi.ac.uk/pub/databases/gencode/Gencode_human/release_XX/gencode.vXX.long_noncoding_RNAs.gtf.gz.

56. Generate the reference genome.

$ STAR --runThreadN 12 \ --runMode genomeGenerate \ --genomeDir GRCh38_star_index \ --genomeFastaFiles GRCh38.primary_assembly.genome.fa \ --sjdbGTFfile gencode.vXX.long_noncoding_RNAs.gtf \ --sjdbOverhang 149

***Note:*** Here, we generate a genome with lncRNA annotations only. If you would like to analyze coding genes as well, repeat steps 55 and 56 with protein coding annotation files, which will generate a protein coding reference genome.

57. Align reads to the reference genome and generate a bam file containing all mapped reads as output. Troubleshooting 1.

$ STAR --runThreadN 12 \ --readFilesIn ath_seed_sample.fastq \ --genomeDir ath_star_index \ --outSAMtype BAM SortedByCoordinate \ --outFileNamePrefix seed_sample \ --outSAMunmapped Within \ --outWigType wiggle

58. Download and install the tool NRSA (Nascent RNA Sequencing Analysis) available on: https://github.com/vermeulenlab/lncRNA/.

***Note:*** The NRSA_guide.txt file includes information about the download and installation of the NRSA tool. The NRSA perl scripts Pause_PROseq.pl will be used for calculating the differential expression of expressed genes. The NRSA contains processed reference files from various organisms. In this protocol, we are using the processed version of the GRC38 reference files which are the same files used for mapping the GRO-seq reads. Ensure that the version of the genome used by NRSA is consistent with the one to which the reads were aligned to in the previous step.

59. To generate output files, execute the following steps sequentially.

***Note:*** In this example, the mapped reads from two different conditions were named 'condition1.bam' and 'condition2.bam'. In the case of multiple BAM files corresponding to different replicates, all BAM files should be added and separated by a space (e.g. -in1 condition1_rep1.bam condition1_rep2.bam -in2 conditions2_rep1.bam condition2_rep2.bam).

$ perl ./bin/pause_PROseq.pl -o ./pause_out/ -in1 ./data/condition1.bam ./data/condition2.bam -in2 ./data/condition2.bam -m GRCh38

$ perl ./bin/eRNA.pl -w ./pause_out/ -in1 ./data/condition1.bam ./dat/condition2.bam -in2 ./data/condition2.bam -m GRCh38

60. Obtain the output files gb_change.txt, which contain transcriptional changes of genes .

***Note:*** These output files can be imported into R, Python, and Microsoft Excel for further analysis. Depending on your experimental design, different cutoffs such as minimum read coverages or specific fold changes (control vs treatment) can be used to define a list of candidate lncRNAs.

### Visualizing expression data and defining target regions

**Timing: 10–20 min per file**

**Timing: 2–10 min per candidate**

Here, we briefly outline the steps to visualize custom expression data on the UCSC genome browser. We further detail how to define and target genomic regions of interest.

***Note:*** Hereafter, we only briefly describe the generation of custom expression data tracks on the UCSC genome browser. Detailed documentation can be found in the User's Guide. Alternatively, the protocol using bigWig files can be found here.

61. Remove any “track” or “browser” lines from your .wig files output obtained in the previous step.
62. Generate bigWig files from your .wig files using the binary utilities fetchChromSizes and wigToBigWig from the UCSC utilities.
63. Host the bigWig files on a http(s) or ftp accessible location (e.g., github).
64. Generate the tracks with the UCSC protocol for bigWig files linked above.

***Note:*** Here, we describe how to select genomic regions of interest for efficient silencing with the CRISPRi system.

65. Open the output matrix obtained from the GRO-Seq data, and use the ENSEMBL gene-IDs to visualize the regions on your UCSC track. A visual example is depicted in Figure 3.

**Figure 3:**
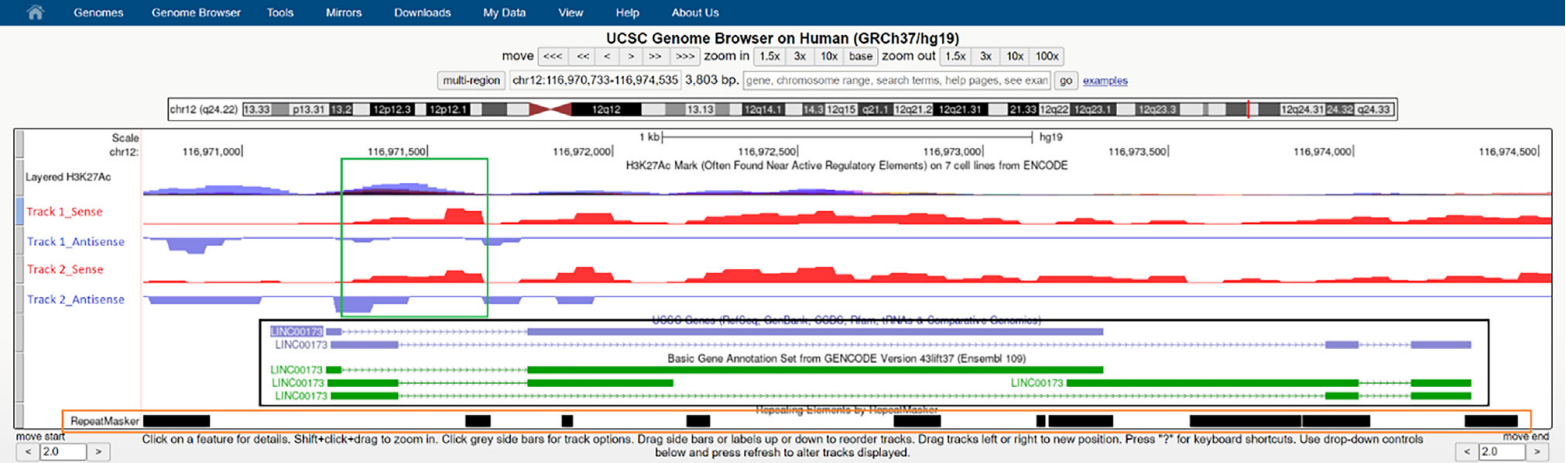
Visualization of GRO-seq data with the UCSC genome browser UCSC genome browser screenshot showing lncRNA transcripts (green\blue annotations) and sequencing tracks of 2 cell lines (track 1 and 2). Read coverage on the sense (red) and antisense (blue) strands are shown as separate tracks. The presence of H3K27Ac (transcriptionally active chromatin) is also displayed. Important features to select the region of interest are highlighted; transcription start site peaks (green box), transcript annotations (black box), and repeat sequences (orange box).
66. Zoom in on the TSS region and define a 400 nucleotides (nts) stretch by selecting 50 nts upstream and 350 nts downstream of the transcription initiation signal. An example is shown in Figure 4.

**Figure 4:**
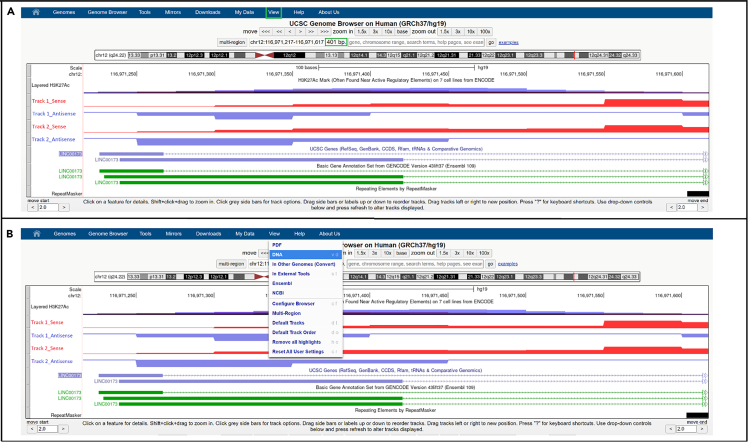
Defining and retrieving target sequences (A) Zoomed in view showing the selection of a genomic region encompassing 50 nucleotides before and 350 nucleotides after the transcription start site. The ”View” button is highlighted in green. (B) The ”View” dropdown menu, with the correct DNA option selected.

***Note:*** In Figure 3, the displayed transcript is encoded on the sense strand (red). The TSS can be defined by locating the beginning of the read coverage for your gene of interest. While annotations can help to pinpoint a region, significant variations can be observed between previously annotated and observed TSS in your specific cell system. When annotations and GRO-seq reads are not perfectly aligned, we recommend to use your sequencing reads to determine the TSS. In addition, most TSSs will also display some transcription (initiation peak) on the opposite strand (here: blue), which is helpful to pinpoint the right region.

67. Following the “Zoom in” on the region of interest, the DNA sequence is retrieved by clicking on “View” and “DNA” (highlighted).
  a. On the new page, check the boxes “Mask repeats” and “to N”, prior to retrieving your DNA sequence by clicking “get DNA”.
68. Copy the sequence and paste it in a new Notepad file, name it according to the ENSEMBL Gene-ID as “ENSG00000XXXXXX.txt” and save it in the CRISPRi folder. It is imperative to save all the sequences separately to properly run the guide design package.

***Note:*** Following these steps, you will have a CRISPRi folder filled with .txt files of all candidate TSS sequences (−50 nts to +350 nts = 400 nt total), named by their ENSEMBL Gene-IDs.

### CRISPRi screen

#### Design gRNAs

**Timing: 2–3 h to prepare the packages/files and 5–15 min for the design of gRNAs (per candidate)**

Here we describe the design of guide RNAs (gRNAs) to epigenetically block the transcription of selected genomic regions using CRISPRi. Troubleshooting 2 & Troubleshooting 3.

69. Download a human genome file by opening up a new terminal window and running:

cd $DIR

sudo wgethttp://hgdownload.soe.ucsc.edu/goldenPath/hg38/bigZips/hg38.fa.gz.http://hgdownload.soe.ucsc.edu/goldenPath/hg38/bigZips/hg38.fa.gz

***Note:*** We recommend using hg38 (from ENSEMBL) as reference genome and bowtie to create an index. To design gRNAs and assess their specificity to one genomic region, we use CCTop^4^^,^^11^ and BLAT, respectively.

70. Unpack the archive to get the file “hg38.fa”. Then, we install all the packages via the terminal, starting with bowtie:

sudo gunzip hg38.fa.gz

sudo apt-get install -y bowtie

71. Then, to install CCTop, run:

pip install CCTop.

72. The installation of BLAT is slightly complex and uses a C compiler to *make* and *make install* the packages. If you don’t have an existing C compiler on your computer, run:

sudo apt install build-essential.

73. Download and install a library that BLAT uses:

wgethttp://downloads.sourceforge.net/project/libpng/libpng16/older-releases/1.6.2/libpng-1.6.2.tar.gz

tar -xvzf libpng-1.6.2.tar.gz

cd libpng-1.6.2

./configure

make

sudo make install

74. Go back to $DIR, download the BLAT source code archive, unpack it, and move all the library files to the /lib/ folder within the BLAT folder:

cd ..

wgethttp://users.soe.ucsc.edu/∼kent/src/blatSrc.zip

unzip blatSrc.zip

cp ./libpng-1.6.2/png.h ./blatSrc/lib/

cp ./libpng-1.6.2/pngconf.h ./blatSrc/lib/

cp ./libpng-1.6.2/pnglibconf.h ./blatSrc/lib/

75. Finally, you have to configure some variables to allow BLAT to run from the terminal:

echo $MACHTYPE

MACHTYPE=x86_64

export MACHTYPE

sudo mkdir -p ∼/bin/$MACHTYPE

make

echo 'export MACHTYPE=x86_64' >> ∼/.bashrc

echo 'export PATH=$PATH:∼/bin/$MACHTYPE' >> ∼/.bashrc

source ∼/.bashrc

76. Run BLAT from the terminal by typing *blat* in the terminal.
77. Open the “.profile” file in your /home/**username**/ directory in notepad and add the following line at the end to ensure you can always use blat from the terminal, and use bash loops:

export PATH=$PATH:∼/bin/$MACHTYPE

78. Create a bowtie index file from the human genome for CCTop. This should output multiple .ebwt files. Execute the following code in $DIR:

bowtie-build -r -f hg38.fa human

79. Run CCTop to design CRISPRi gRNAs on one of our candidate sequences (“$FILE.txt”):

cctop --input $FILE.txt --targetSize 19 --index human

80. You can also run all the files automatically by running:

for SAMPLE in ∗.txt; do cctop --input ${SAMPLE} --targetSize 19 --index human; done

81. The output should consist of 3 files per input candidate: a .bed file with gRNA locations along your input sequence, a .fasta file with all designed gRNA sequences, and a .xls file with extra information, including the CRISPRater score for each gRNA.
82. Create new directories for the different output files and move all files to these directories. In addition, the hg38.fa file is required for BLAT and should therefore be moved to the fasta folder as well:

sudo mkdir fasta

sudo mkdir xls

sudo mv ∗.fasta ./fasta

sudo mv ∗.xls ./xls

sudo mv hg38.fa ./fasta

***Note:*** We highly recommend to create a 11.ooc file before running BLAT, as it will significantly speed up your query. If you want to do so, run:

cd fasta

blat hg38.fa /dev/null/ /dev/null/ -makeOoc=hg38.fa.11.ooc -repMatch=1024

83. Blat all files by making a new output directory and by running:
  sudo mkdir Blat
  for i in ∗".fasta"; do echo "{$i}"; name=${i%%.fasta}; blat hg38.fa "${i}" -ooc=hg38.fa.11.ooc -minIdentity=90 -minScore=15 -minMatch=1 -oneOff=1 -out=blast8 ./Blat/"${name}".psl; done
  a. This should generate a .psl file, which includes all gRNAs targeting a specific candidate region. Every file will be named according to their specific ENSEMBL gene-ID.
    ***Note:*** We recommend to covert the .xls output file into a .csv file, which facilitates subsequent steps in python. To this end, we use the libreoffice-calc package from the terminal.
84. Install the libreoffice-calc package if necessary.

sudo apt install libreoffice-common

sudo apt install libreoffice-calc

85. Convert the .xls files to .csv from the “xls” folder, and move them back to the main directory.

***Note:*** libreoffice can only handle 250 files at a time, so if you have more candidates, move them to separate folders and execute the code in that folder.

86. Continue from step 83:

cd ..

cd xls

libreoffice --headless --convert-to csv ∗.xls

sudo mv ∗.csv ..

(If running from separate folders for >250 candidates, use sudo mv ∗.csv ../..)

***Note:*** To rearrange the .csv file, we recommend to create a folder called “Clean”, and open the “Ordered csv.html” file. Execute this code in jupyter notebook, and make sure to substitute all the correct file paths (“./” is the current directory of the .ipynb file).

87. Start jupyter notebook by running the following from $DIR:

jupyter notebook

***Note:*** If you are running Ubuntu via WSL, add the command --no-browser.

88. Now you should have an organized .csv file in the “Clean” folder for every candidate, as well as a BLAT .psl file in the “fasta/Blat/” folder.
89. Create a new folder called “Final” in the CRISPRi main folder and run the code from “Cleanup and guide picking.html” in jupyter notebook.

***Note:*** Make sure to substitute all the correct file paths and to adapt the “preseq” and ”postseq” variables according to the homology arm sequences present in your selected gRNA vector.

The end result is the “Merged.csv” file, containing all selected gRNA sequences, both with and without homology arms (“OrderSeq”).

#### Stable genomic integration of KRAB-dCas9

**Timing: 5 days**

Here we describe the transduction of cell lines to stably integrate an inducible CRISPRi system. In this protocol, we use an inducible KRAB-dCas9 system or the TRE-KRAB-dCas9-IRES-BFP plasmid (Addgene #85449). This construct allows for dox-dependent expression of KRAB-dCas9 and BFP, which enables a more controlled setting as compared to constitutively active promoters. The dox-dependent induction of KRAB-dCas9 and BFP requires the co-expression of the reverse tetracycline transcriptional activator (rtTA). Stable integration and constitutive expression of rtTA is achieved using pHR-EF1Alpha-Tet-on 3G (Plasmid #118592). Cell populations that properly induce KRAB-dCas9/BFP (cells co-transduced with KRAB-dCas9/BFP and rtTA) are enriched by FACS sorting. For an overview of all preparatory steps, see Figure 6A.

**CRITICAL:** This protocol assumes a basic understanding of the production of lentiviral particles (and associated biosafety procedures) and 2D cell culturing. A general protocol to generate lentiviral particles can be obtained here.

#### Day 1

**Timing: 1 h**

90. Split cells of interest and seed approximately 4–6 x10^5^ cells (∼70%–80% confluent) in 1 mL of medium per well of a 6-well plate.

***Note:*** The amount of cells depends on the growth rate and size of your cells.

91. Thaw the pre-generated viruses (filtered 0.45 μm) OR harvest fresh lentiviruses containing KRAB-dCas9/BFP and rtTA.
  a. Add 1 mL of virus to the cell suspension and polybrene at a final concentration of 4 μg/mL.
  b. Centrifuge the plate for 30 min at 500 × *g* (32°C).
  c. Place the plate in a cell culture incubator for 14–18 h at 37°C.

***Note:*** In our experience, using 1mL of lentiviral supernatant (not concentrated) is sufficient to obtain a transduction efficiency that ranges between 1% and 10%. However, concentrating lentiviral supernatants may be necessary to efficiently co-transduce certain cell types.

#### Day 2

**Timing: 30 min**

92. Refresh the medium.

#### Day 3–5

**Timing: 30 min**

93. Transfer cells into a bigger vessel when the confluency reaches >90%.
94. Maintain and expand cells at an appropriate confluence in at least 2 separate cell culture vessels.

### Enriching cells harboring the inducible CRISPRi system

**Timing: Several weeks**

This section describes the selection of transduced cells by fluorescence activated cell sorting (FACS). Cells co-transduced with TRE-KRAB-dCas9-IRES-BFP plasmid (Addgene #85449) and EF1Alpha-Tet-on 3G (Plasmid #118592) are dox-treated and BFP+ cells are sorted. To gradually enrich cells that properly induce KRAB-dCas9/BFP, multiple FACS sorting cycles are required. In each cycle, cells are dox-treated, FACS sorted (BFP positive cells sorted) and cells expanded (without dox). Enrichment cycles are performed until the vast majority of cells (>95%) can induce the BFP expression in a dox-dependent manner. In addition, leaky cells can be removed by sorting out BFP positive cells in absence of dox.

***Note:*** Our experimental setup uses a construct that allows the selection of cells by a fluorescent marker. Other constructs may contain distinct selection markers and may require a different selection approach.

#### Day 6

**Timing: 5 min**

95. Add dox to the culture medium (final conc. 1 μg/mL).
  a. Incubate the cultures at least 24h to induce the BFP expression.
  b. Keep one cell culture vessel without dox to use as control sample for the FACS.

***Note:*** In this protocol, we use 1 μg/mL of dox in the culture medium to induce the CRISPRi system. However, different cell lines may show different sensitivity to doxycycline, which may require titration experiments to establish ideal experimental conditions.

#### Day 7

**Timing: 2–3 h**

96. Trypsinize and collect cells into a 15 mL tube.
  a. Spin down for 5 min at 500 × *g*.
  b. Remove the supernatant and resuspend the cell pellet in 1–2 mL of medium (depending on pellet size).
  c. Prepare a 15 mL collection tube for sorting with 2 mL of medium.

***Note:*** In this protocol, we performed cell sorting using the SH800 Cell Sorter (Sony). Settings may differ if other cell lines and other FACS machines are used. An untreated control sample (no dox) is essential to properly gate BFP+ cells.

97. Determine the gating. An exemplary gating strategy is depicted in Figures 5B–5F.
  a. To gate living cells, use the forward (FSC) and backward scatter (BSC, equivalent to side scatter SSC) area (A).
  b. Select singlets by using an FSC-height and width density plot.
  c. BFP+ cells are sorted using the 405 nm laser.

**Figure 5:**
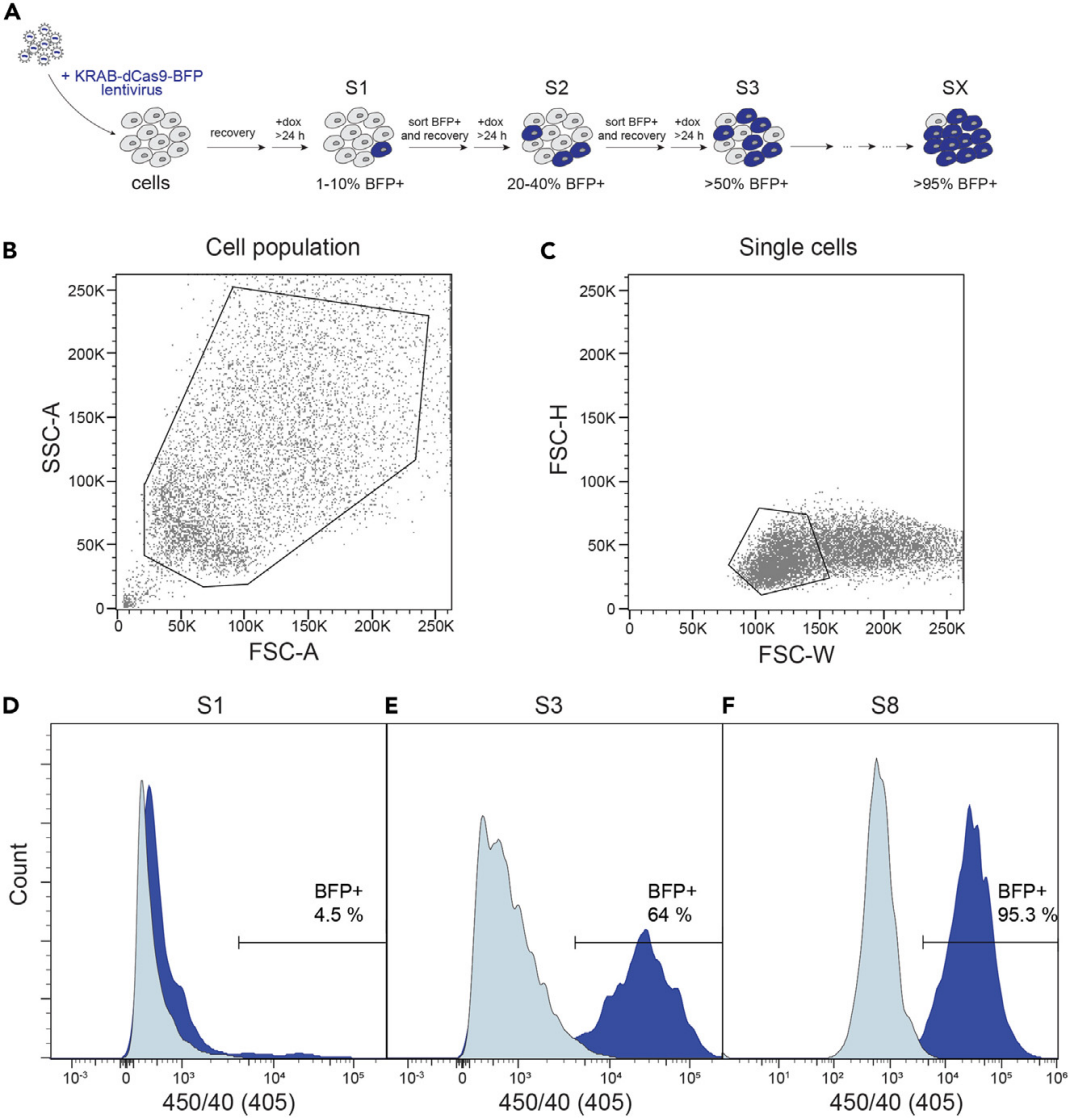
Generation of dox-inducible KRAB-dCas9-BFP cells (A–F) (A) Schematic illustrating steps to generate CRISPRi cell line using FACS sorting. Gating strategy is shown in (B) and (C). Enrichment of BFP+ cells (dark blue) following 1 (D), 3 (E) and 8 (F) sorting cycles. Uninduced cells (light blue) were used as negative control.
98. Sort the BFP+ cell population into the prepared collection tube.
  a. Transfer sorted cells back into an appropriate cell culture vessel depending on the number of sorted cells.

#### Day 8–14

**Timing: 2–5 h**

99. Culture and expand the sorted cells accordingly.

***Note:*** Depending on the transduction efficiency and proliferation rate of your cell line, dox-induction and sorting steps may be performed with an alternative or better suited time-schedule. Alternatively, single cell (BFP+) clone could be sorted and expanded. However, molecular features of single cell clones may not properly represent the complexity or heterogeneity of the initial population.

#### Day 15-day x

**Timing: Several weeks**

100. Repeat steps 95 to 99 until you obtain a homogeneous population (>95% BFP + cells; Figures 5D–5F).

***Note:*** We recommend testing the functionality of your CRISPRi cell line, by targeting an essential gene. This allows to validate the transcriptional repression of a target gene and assess potential cellular phenotypic changes.

### Setting up and performing a CRISPRi dropout screen

**Figure 6:**
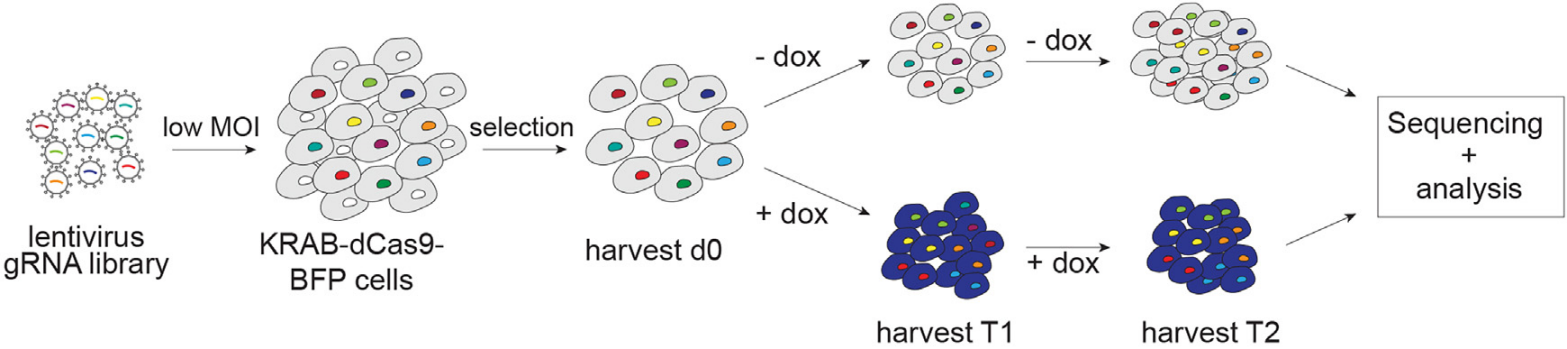
CRISPRi dropout screen layout The cartoon shows key steps required to perform a CRISPRi dropout screen. Following the transduction of a gRNA library in a CRISPRi cell line (KRAB-dcas9 BFP cells), cells are treated or not with dox and collected at various time points (d0, T1 and T2). Sequencing is performed to evaluate the relative abundance of each gRNA across collected samples.

In this section we discuss how to perform a CRISPRi-based dropout screen with adherent cells. An overview of the CRISPRi-dropout screen is shown in Figure 6.

The gRNA library is first cloned into a suited backbone vector, which allows for the generation of lentiviral particles. Then, cells containing the inducible KRAB-dCas9-BFP are transduced with lentiviral particles containing the gRNAs. This should be performed at a low MOI to minimize the presence of multiple integrations per cell. This section describes the titration to obtain a low MOI.

**CRITICAL:** This section assumes a basic understanding of cloning. We followed the protocol by Wang et al.^12^ to clone our gRNA library into a vector backbone and do not further describe these steps here nor are these considered in the estimated timing. We assume a general understanding of lentiviral particles production (and associated safety procedures) and 2D cell culturing. Troubleshooting 4.

***Note:*** We used the pDECKO-mCherry backbone (Addgene #78534) to clone our gRNA library, a vector that contains the mCherry fluorescent protein as well as a puromycin resistance gene for selection. We use the live cell labeling with mCherry to evaluate the transduction efficiency by flow cytometry and to adjust the conditions (e.g., cell density and amount of virus) to obtain a low MOI. Then, we select transduced cells by adding puromycin. Selection of cancer cell lines is usually completed within 2 weeks. We highly recommend using both the antibiotic-based selection as well as a fluorescent marker to easily control and complete these steps. Prior to transduce CRISPRi cell lines, the pDECKO-gRNA library DNA preparation can be PCR amplified (see section “Preparing sequencing libraries”) and sequenced to validate the presence of each gRNA (optional).

#### Day 1

**Timing: 1 h**

101. Harvest cell cultures and seed 5 × 10^6^ cells per T75 flask.
  a. Prepare at least 4 flasks.
  b. Keep one without virus as control.
102. Thaw pre-generated virus.

***Note:*** Number of cells may vary depending on the features (e.g., size and proliferation) of your cells of interest. In general, 70%–80% seeding confluency will yield robust and reproducible results. We usually generate a big volume of viral particles containing the gRNA library to avoid multiple titrations and minimize possible batch effects. We filter, aliquot and store the virus suspension at −80°C. Lentiviral particles can be stored several months without significant impacts on the transduction efficiency.

103. Add different amounts of virus per flask, e.g., 100, 400 and 800 μL of virus.
104. Add polybrene at a concentration of 4 μg/mL to each flask.
105. Place the flasks for 14–18 h at 37°C in a cell culture incubator.

#### Day 2

**Timing: 30 min**

106. Refresh the medium.

#### Day 3

**Timing: 2–3 h**

107. Harvest and collect cells in tubes suited for flow cytometry analysis.

***Note:*** We acquire the samples on the SH800 Cell Sorter (Sony) to assess transduction efficiencies. Settings may differ if other cell lines, plasmids and FACS machines are used.

**Figure 7:**
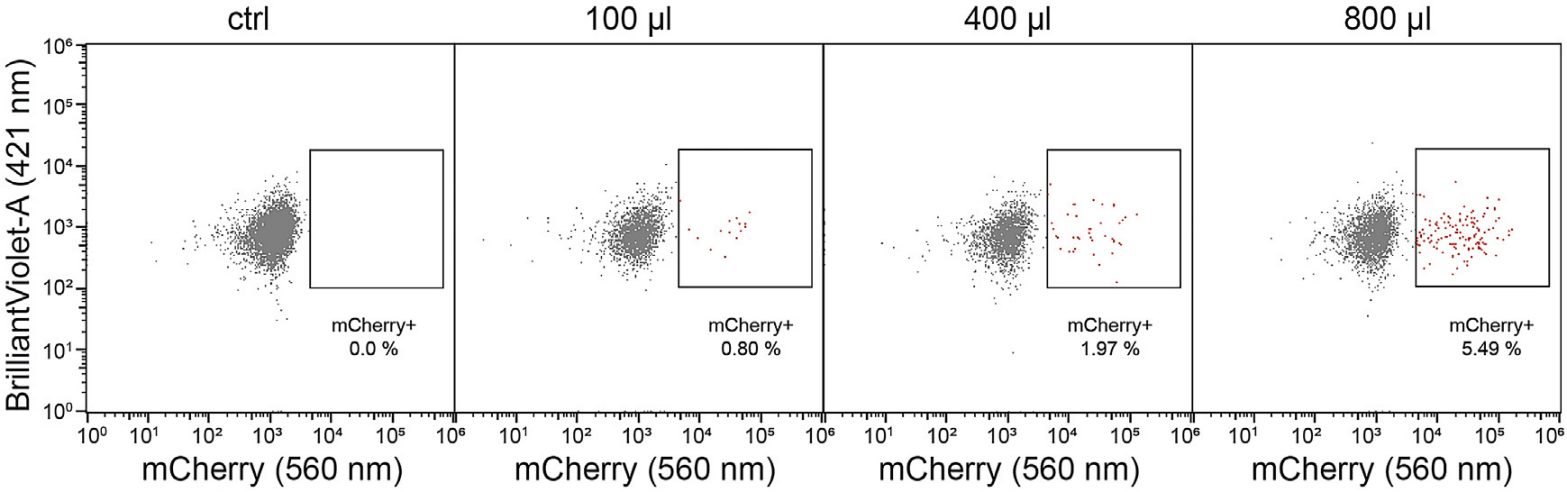
Titration of viral particles harboring the gRNA library CRISPRi-engineered cells were either untransduced (ctrl) or transduced with an increasing amount of lentiviral particles (100, 400 and 800 μl). The transduction efficiency (percentage of mCherry+ cells) is displayed for each condition by plotting the mCherry signal (x-axis) against the BrilliantViolet signal (y-axis).

108. Setup the gating strategy as mentioned in step 97.
  a. Visualize mCherry+ cells by using the 561 nm laser. An untransduced control sample is required to properly gate mCherry+ cells.
  b. Examples of transduction efficiencies following the incubation of CRISPRi-engineered cells with an increasing number of lentiviral particles harboring the gRNA library (mCherry+) is shown in Figure 7. Troubleshooting 7.

### Transduction and selection of the gRNA library

Here we detail the steps leading to the transduction and selection of cells harboring the gRNA library.

The library is transduced with low MOI (<0.5) and cells are selected with the appropriate marker (e.g., Puromycin or Blasticidin). The screen is usually initiated with a gRNA coverage of 500× (e.g., 1,000 gRNA library requires >500,000 cells) for each replicate and in presence or absence of dox. We recommend harvesting several time points to follow dropouts over time.

**CRITICAL:** To ensure a good representation of each gRNA, we usually transduce the cells of interest with a 200× coverage. Screens can also be initiated with a coverage of at least 200×. However, we do recommend using a coverage of 500× or more if possible.

#### Day 4

**Timing: 1–2 h**

109. Seed a sufficient amount of T75 flasks with each ∼5 × 10^6^ cells (70%–80% confluent).

***Note:*** The required number of flasks should be determined based on the size of your library and the titration of your lentiviral suspension. We usually aim to cover every gRNA 200 times with a transduction efficiency varying between 1% to 10%.

110. Thaw pre-generated virus.
111. Add an adequate amount of virus per flask to obtain a transduction efficiency ranging between 1% to 10% based on previous titration (e.g., 800 μL would achieve ∼5% mCherry+ cells in Figure 7).
112. Add polybrene at a concentration of 4 μg/mL to each flask.
113. Place the flasks for 14–18 h at 37°C in a cell culture incubator.

#### Day 5

**Timing: 30 min**

114. Refresh the medium.

#### Day 6

**Timing: 1 h**

115. Maintain and expand your cells at an appropriate confluency.
  a. Transfer cells into a bigger vessel when they reach confluency.

#### Day 7

**Timing: 1 h**

116. Start the selection of transduced cells by adding puromycin to the culture medium.

***Note:*** For CRC cell lines, we used concentrations ranging between 1 and 5 μg/mL of puromycin, depending on their sensitivity. Alternatively, determine the amount of puromycin needed to kill untransduced cells by performing a dose-response experiment. Selection of cancer cell lines is usually completed within 2 weeks.

#### Day 8 – X

**Timing: 1–4 weeks**

117. Maintain and expand the cell cultures at an appropriate confluency.
118. Keep selecting cells with puromycin until the cell population is color-labeled (> 95% mCherry+).
119. Verify the successful selection by flow cytometry.

***Note:*** When maintaining the cell culture, it is important to preserve the complexity of the gRNA library by seeding cells with at least 200× coverage.

### Dropout screen initiation and time point collection

**Timing: 7–30 days, depending on experimental setup and harvesting time points.**

Here we describe the conditions and steps required to initiate and complete a CRISPRi dropout screen.

**CRITICAL:** We initiate our CRISPRi screens with >500× coverage and recommend to use at least 200× for each replicate. We also advise to freeze a stock of cells containing your gRNA library that contains at least a 200× coverage per vial. However, attention should be paid during the thawing step, as important cell death may impact the complexity and therefore skew the representation of gRNAs.

***Note:*** To monitor cell population dynamics, we recommend harvesting 3 time points (e.g., 0, 12 and 20 days).

#### Day 1

**Timing: 1–2 h**

120. Harvest all the library-transduced cells and collect them in a 50 mL falcon.
  a. Count the concentration of cells.

***Note:*** For better accuracy, ensure that trypsinized cells are fully singularized before counting.

121. For the “day 0” samples, collect in 3 different tubes (3 replicates) a number of cells corresponding to at least 200× the size of the gRNA library.
  a. Centrifuge the cells for 5 min at 500 × g and remove the supernatant.
  b. Store cell pellets at −20°C.
122. For the screen, seed an adequate number of cells and culture vessels to reach a library coverage of at least 200× for each replicate.
  a. Choose ideal culture vessel and cell seeding density to accommodate 4 days of cell expansion without reaching confluency (>95%).

***Note:*** Depending on the size of your library or properties of your cell line, it might be necessary to seed cells in multiple plates per replicate. We recommend performing 3 replicates in parallel with and without dox to obtain a robust statistical analysis of the screen.

123. Add dox to a final concentration of 1 μg/mL (or use the ideal dox concentration for your cell line as mentioned above) to the 3 induced (+) replicates.
  a. Maintain the other 3 replicates untreated (-).
  b. Label the culture vessels appropriately.
  c. Place them back in a cell culture incubator at 37°C.

#### Day 3

**Timing: 10 min**

124. Refresh the medium of all culture vessels.
  a. Add dox (1 μg/mL) to induced (+) culture vessels to maintain a sufficient expression of KRAB-dCas9.

#### Day 5

**Timing: 2–3 h**

***Note:*** To maintain viable conditions for the cells, we replate cultures every 4 days over the course of the screen.

125. Trypsinize cells.
  a. Collect each replicate in separate and labeled 50 mL tubes.
  b. Count the cells.
  c. Seed back cells into new culture vessels as performed in step 122.
  d. Add 1 μg/mL dox to the medium of induced (+) culture vessels.
126. Repeat step 124 every 2 days and step 125 every 4 days until the first time point (T1) is reached.

#### Day X: First time point (T1)

**Timing: 2–4 h**

127. To collect timepoint samples, trypsinize cells.
  a. Collect each replicate in separate and labeled 50 mL tubes.
  b. Count the cells.
128. For each replicate, transfer a number of cells corresponding to at least 200× the size of the gRNA library to a labeled 15 mL tube.
  a. Centrifuge cells for 5 min at 500 × *g*.
  b. Remove the supernatant.
  c. Freeze the pellets at −80°C.
129. If another time point (TX) is harvested, seed back cells into new culture vessels as performed in step 122.
130. Repeat step 124 every 2 days and step 125 every 4 days until the next time point (TX) is reached.
131. **Day Y**: Next time point
132. Perform steps 127 to 130.

### Preparation of sequencing libraries

**Timing: 2 days**

To prepare sequencing libraries, we PCR-amplify gRNAs (PCR1) from gDNA obtained from collected samples. In a second amplification step (PCR2), adapters and indices are added to perform standard Illumina multiplex sequencing. PCR primers used to generate sequencing libraries are shown in Table S2.

***Note:*** We do not describe the steps required for the library quantification. However, a detailed protocol can be found online at the manufacturer’s page: NEBNext® Library Quant Kit for Illumina®.

133. Extract the genomic DNA from each cell pellets with Genomic DNA Purification Kit or a similar product.
134. PCR-amplify gRNA sequences using PCR1 primers, which are specific to the vector backbone.

***Note:*** To properly amplify the complexity of a library by PCR, each gRNA should be represented by at least 200 genomes (one integration per genome). Considering that the human diploid genome is approximately 6.5 pg, then a library of 10,000 gRNAs requires to PCR-amplify ∼13 μg of gDNA (200 × 10,000 × 6.5pg).

135. PCR-amplify amplicons from PCR1 with PCR2 primers, in order to add standard Illumina adapters.

***Note:*** The second PCR is also used to add indices to samples, thus allowing multiplex sequencing. For details, see Table S2.

136. Purify amplicons obtained from PCR2 from the gel using the NucleoSpin Gel & PCR Clean-up kit or similar.
137. Quantify the concentration of all libraries, using the NEBNext® Library Quant Kit for Illumina®.
138. Dilute samples to the desired concentration (usually between 20–50 nM) and pool all libraries (if multiplex sequencing is performed).

***Note:*** To improve accuracy, we recommend an initial dilution of libraries at a higher (2×) concentration (40–100nM). Quantification is then repeated on the “2×” diluted samples before the final dilution.

139. Sequence all samples using next-generation sequencing.

***Note:*** Using single-end 50 bp sequencing reads (SE50) is sufficient to retrieve your gRNA sequences. We also recommend adding 5% phix and to cover each gRNA with at least 200 reads.

### Screen analysis

**Timing: 30 min to install and prepare, and 30–60 min per 30 million reads (for step 140)**

**Timing: 30–60 min per 30 million reads (for step 147)**

Here we describe how to analyze the sequencing data obtained from a CRISPRi dropout screen. We use the MAGeCK package to count the reads and DESeq2 package for subsequent analyses. Specific cutoffs and filters are also applied to the data to produce a list of dropout candidates. Troubleshooting 2 & Troubleshooting 3.

**CRITICAL:** The analysis is executed and tested on an Ubuntu (version 20.04) virtual environment via Windows Subsystem for Linux (WSL, version 1).

**Before you start:** Download the .fastq files generated by the dropout screen, and place them in a convenient directory (“$DIR” from now on). If you performed paired-end sequencing, only the forward reads (_R1 or similar) are necessary, therefore place the reverse reads (_R2 or similar) in a separate folder in $DIR. Download and install R (The Comprehensive R Archive Network (r-project.org)) on your system. We highly recommend R studio for writing and executing R codes.

Prior to obtaining the read counts for each gRNA, adapter sequences flanking the gRNA insert must be trimmed. The following steps are adapted from the documentation of Trimmomatic.

140. To install Trimmomatic, execute the following commands in a terminal window:

cd $DIR

wgethttp://www.usadellab.org/cms/uploads/supplementary/Trimmomatic/Trimmomatic-0.36.zip

unzip Trimmomatic-0.36.zip

sudo mkdir ./Trimmed

***Note:*** In order to run Trimmomatic, Java should be installed on your system. If Java is not present, run:

sudo apt install default-jre

sudo update-alternatives --install "/usr/bin/java" "java"

java –version

141. Identify the gRNA flanking sequences by running the following code on one of your .fastq output files (“$FILE”)

cd $DIR

head $FILE.fastq

Example output:

**Figure:**
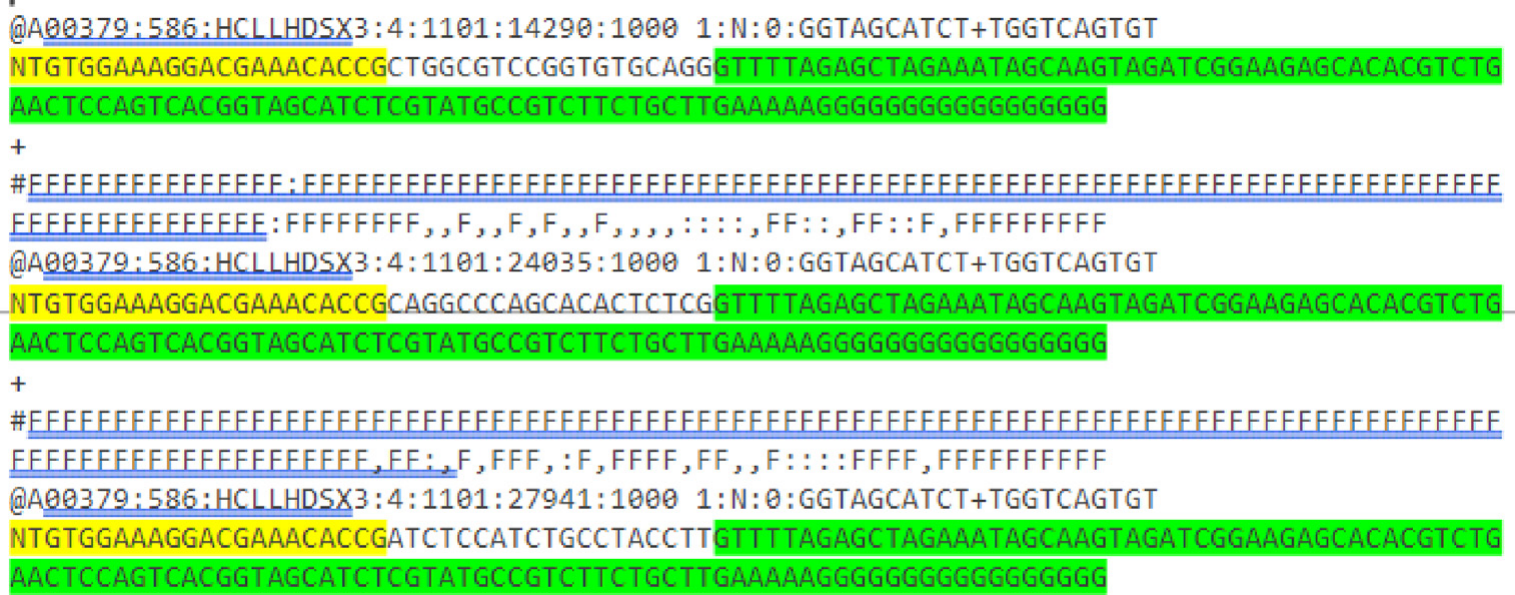

***Note:*** Adapter sequences before (yellow) and after (green) the gRNA are highlighted for clarity.

142. Count the length of the adapter that prepends the gRNA inserts (in this case, 22 nucleotides).
143. Pre-process the reads by running the following command.
  a. Replace $FILE with your filenames
  b. Replace the numbers following “-threads” and “HEADCROP:” with your specific number of cores and the length of the adapter determined in step 142, respectively:

java -jar ./Trimmomatic-0.39/trimmomatic-0.39.jar SE -threads 16 $FILE_trim.fastq ./Trimmed/$FILE_Trim.fastq HEADCROP:22 CROP:19

144. Run all the files automatically by executing a bash loop in the terminal.

for SAMPLE in ∗.fastq; do java -jar ./Trimmomatic-0.39/trimmomatic-0.39.jar SE -threads 16 ${SAMPLE}.fastq ./Trimmed/${SAMPLE}_Trim.fastq HEADCROP:22 CROP:19; done

145. Verify successful cropping of the reads by running:

cd ./Trimmed

head $FILE_Trim.fastq

146. Output:

A00379:586:HCLLHDSX3:4:1101:14290:1000 1:N:0:GGTAGCATCT+TGGTCAGTGT

CTGGCGTCCGGTGTGCAGG

+

FFFFFFFFFFFFFFFFFFF

@A00379:586:HCLLHDSX3:4:1101:24035:1000 1:N:0:GGTAGCATCT+TGGTCAGTGT

ACAGGCCCAGCACACTCTC

+

FFFFFFFFFFFFFFFFFFF

@A00379:586:HCLLHDSX3:4:1101:27941:1000 1:N:0:GGTAGCATCT+TGGTCAGTGT

ATCTCCATCTGCCTACCTT

***Note:*** The result is a cropped version, containing only the guide sequence.

147. Next, we use MAGeCK to count the reads. To install MAGeCK, open a new terminal window and run:

sudo wgethttps://sourceforge.net/projects/mageck/files/0.5/mageck-0.5.9.4.tar.gz

tar xvzf mageck-0.5.9.4.tar.gz

cd mageck-0.5.9.4

python setup.py install

***Note:*** Other packages similar to MAGeCK (e.g. BAGEL^13^ and JACKS^14^) could also be used to perform this step.

148. Test by typing mageck in the terminal, which should output a help prompt for using MAGeCK.
149. Prepare the library file by opening a new excel sheet and adding three columns. An example is shown in Table 5.

**Table 5:** Example of a MAGeCK library file

seqID	gRNAs	Targetgene
1	GCGGGCGAGGTGCCCAAGG	ENSG0000260852.1
2	CAGGCTACCTAGCCTCTCC	ENSG0000260852.1
3	CAGTGTTCCTTAAGAATGG	ENSG0000260852.1
4	ACTAGTAGTCGATCTTACG	ENSG0000260852.1
5	CAAGCAAGATGGCGGAAGA	ENSG0000260852.1
6	GACGAGTAGAAATGGCAGC	ENSG0000260852.1
7	ATGGACTGCGACTCCCGTC	ENSG0000260852.1
8	CACGCGGAGGGTTGTGGAC	ENSG0000260852.1
9	TGGGAAGGAGGTCGGGTCG	ENSG0000260852.1
10	TGCTCAACACCGACTGGAG	ENSG00000163597.14
11	ATGGTAAGGCTGCCTGGGT	ENSG00000163597.14

Labeling: “seqID” is an identifier for the guide; “gRNAs” is the guide sequence; “targetgene” is the gene targeted by the gRNA.
150. Save this excel sheet as a .csv in the /$DIR/Trimmed directory.
151. To count all the files, run the following command in the /$DIR/Trimmed directory ($FILE is the library file.

mageck count –l $FILE.csv --fastq ∗.fastq

152. This should output multiple files, including .count.txt and .count_summary.txt. Troubleshooting 5.

**Figure 8:**
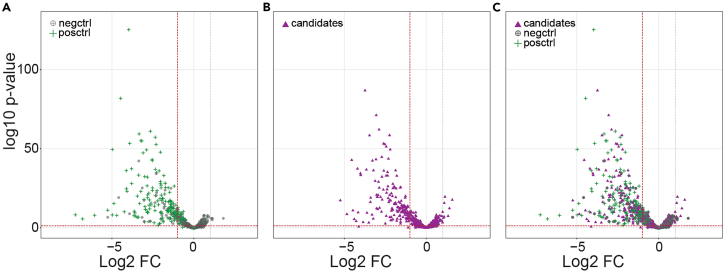
Dropout screen analysis (A–C) The dropout fold changes (log2 FC) of (A) negative controls and positive controls, and (B) candidate-targeting gRNAs (endpoint vs. day 0) are plotted against the p-value (log10 p-value). Plot (C) shows the overlay of plots (A) and (B).

***Note:*** We recommend to review the counting process by constructing a volcano plot from your count data (see Figure 8). It is important to verify that the negative controls are not dropping out, while the positive controls are. To generate volcano plots, we recommend using the R package EnhancedVolcano.

#### Identifying dropout hits

**Timing: 1 h (for step 153)**

In this section we describe a general approach to identify potential dropout hits. To this end, we compare the relative presence of each gRNA in induced (+) and untreated (-) samples, using the DESeq2 package in R. Troubleshooting 6.

153. Install and load DESeq2 in R via Bioconductor by running the following commands:

if (!require("BiocManager", quietly = TRUE))

install.packages("BiocManager")

BiocManager::install("DESeq2")

library(DESeq2)

154. Format the data to remove the sgRNA column from the count matrix output. Execute in R:

data <- read.delim(“$DIR/Trimmed/.count.txt”, sep=”\t”)

data = subset(data, select=-c(sgRNA))

write.csv(data, “$DIR/Trimmed/count_formatted.csv”, row.names=FALSE)

155. Specify which samples belong to which condition in the count matrix. The sample names and their original names can be found in .countsummary.txt.
  a. Name the samples that were induced (+) “treated”
  b. Name the control samples (-) “untreated”. Using R:

name<-c("sample1","sample2","sample3","sample4","sample5","sample6","sample7","sample8","sample9","sample10","sample11","sample12","sample13","sample14")

condition <- c("untreated", "untreated", "untreated", "treated", "untreated", "treated", "untreated", "treated", "untreated", "treated", "untreated", "treated", "untreated", "treated")

df <- data.frame(condition, row.names=name)

156. Load all files in R.
  a. Turn all row names into unique values
  b. Cast columns are as the “factor” type
  c. Remove possible whitespace characters from all values in the count matrix
  d. Format the count matrix into a DESeqDataSet:

cts <- as.matrix(read.csv("/$DIR/Trimmed/count_formatted.csv",sep=","))

rownames(cts) <- make.names(cts[,1], unique=TRUE)

cts = subset(cts, select=-c(Gene))

df$condition <- factor(df$condition)

cts <- cts[, rownames(df)]

cts <- as.data.frame(apply(cts,2,function(x)gsub('\\s+', '',x)))

cts[,] <- sapply(cts[,], as.numeric)

dds <- DESeqDataSetFromMatrix(countData = cts,

colData = df,

design = ∼ condition)

157. Run the DESeq function to analyze treated vs. untreated.

dds$condition <- relevel(dds$condition, ref = "untreated")

ddsseq <- DESeq(dds)

158. To identify gRNAs that show a significant downregulation, we recommend starting the analysis with a p < 0.05 and a Log2FoldChange (LFC) < −1.0 as cutoff:

res05 <- results(ddsseq, alpha=0.05)

resdown <- res05[which(res05$log2FoldChange < -1.0),]

***Note:*** The resulting data frame shows gRNAs that are significantly dropping out in the screen. We recommend narrowing down the list of candidates by selecting transcripts targeted by at least 2 functional gRNAs.

159. Make the row names for each target gene identical
  a. Count all the occurrences of identical genes in a new column called ‘good_sgRNAs’.
  b. Filter the data frame to only include genes which have more than 1 ‘good sgRNA’
  c. Save it as a .csv in $DIR:

rownames(resdown) <- gsub("\\..∗","",rownames(resdown))

resdown$good_sgRNAs <- as.numeric(ave(rownames(resdown), rownames(resdown), FUN = length))

candidates <- resdown[which(resdown$good_sgRNAs > 1),]

write.csv(as.data.frame(candidates), file=‘$DIR/candidates.csv”)

160. This should output a list of all genes for which at least 2 gRNAs drop out with an adjusted p-value of < 0.05 and an LFC < −1.0.

## Expected outcomes

The major outcomes of this protocol are lncRNAs that are likely essential for cell proliferation or survival. However, further experiments to validate screening hits and explore their modes of action will still be required. This method provides a fast, high-throughput and efficient way to test hundreds to thousands of putative candidates, making it ideal to identify essential transcripts.

## Limitations

Dropout screens are mostly limited to the identification of transcripts influencing cell proliferation or survival in a 2D culture system. Of note, CRISPRi allows epigenetic silencing of a targeted locus. While the aforementioned strategy maximizes specific targeting, nearby genomic regions may also be influenced and yield “false” positive dropouts. Therefore, we recommend validating any hits with an alternative silencing method (e.g., siRNAs or shRNAs).

## Troubleshooting

### Problem 1

Generation of output matrices: GRO-Seq reads mostly cover ribosomal RNAs.

### Potential solution

One critical step in the GRO-seq procedure reside in the capture of nascent transcripts (harboring Br-UTP) with agarose-conjugated anti-BrdU beads. To reduce rRNA contamination, extra washing steps can be added to the proposed procedure. Alternatively, anti-BrdU pull-down can be performed twice by repeating steps 20 to 51. Certain cell lines have low transcriptional output and, thus, changing the selected cell line could also help to reduce the rRNA contamination.

### Problem 2

Generation of output matrices, design CRISPRi gRNAs & Identifying dropout hits: Running commands from the terminal gives “command not found” output.

### Potential solution

Verify if the package/command that you are running is in the $PATH variable of the system by running “echo $PATH” . If the directory of the binary you want to run is not in the list, you can add it using “export PATH = $PATH:path/to/binary/”.

### Problem 3

Generation of output matrices, design CRISPRi gRNAs & Identifying dropout hits: Permission denied during any step of the guide design or analysis.

### Potential solution

Use sudo chmod and/or chown in the terminal to give permissions and/or take ownership of the files and folders in question.

### Problem 4

Titration of viral particles: Low number of bacterial colonies during the library cloning.

### Potential solution

Many small issues can cause unsuccessful library cloning. However, in our experience, the electroporation step is usually the most critical step. Make sure no air bubbles are introduced in the electroporation cuvette, when transferring the bacterial/library suspension. In our hands, a time constant between 3.5 and 5 ms are usually yielding a good number of colonies.

### Problem 5

Screen analysis: Not all gRNAs are found back after sequencing.

### Potential solution

If more than 1% of gRNAs is not detected in your screen analysis, we recommend sequencing the library DNA preparation used to generate the lentivirus particles. This will determine whether all gRNAs are present in the library (as suggested before step 101). If not, repeating the library amplification and cloning steps is recommended. If the library is complete, then loss of complexity may occur during the virus production and/or the transduction step. To tackle this problem, more HEK293T cells can be used to produce a greater amount of virus particles. In addition, the transduction step should be carefully monitor by facs (using % of mcherry positive cells) to determine that enough cells were transduced to fully represent the library 200×.

### Problem 6

Identifying dropout hits: Dropout screen does not yield many hits.

### Potential solution

Some cell lines may be more resilient and, thus, require a longer period of time to show sensitivity to certain gRNAs. Harvesting more time points or using other cell lines can help to reduce this problem. Alternatively, cell confluency throughout the screen can impact phenotypic changes. Make sure that cells do not reach confluency (>95%) too quickly. If that is the case, seed the required number of cells in more culture vessels.

### Problem 7

Titration of viral particles: Library virus titration does not yield >1% transduction efficiency.

### Potential solution

Verify that your packaging vectors (e.g., PAX2 and VSVG) are working properly by testing the virus production of other transfer plasmids. Cell confluency (too confluent or too sparse) can also heavily influence your transduction efficiency. We recommend testing multiple cell densities in combination with different amount of viral particles. Freeze/thaw cycles of your viral suspension can also affect the structural integrity of your viral particles and, thus, the transduction efficiency. We recommend storing small volumes of viral suspension.

## Resource availability

### Lead contact

Further information and requests for resources and reagents should be directed to and will be fulfilled by the lead contact, Nicolas Léveillé (n.leveille@amsterdamumc.nl).

### Materials availability

This study did not generate new unique reagents.

## Data Availability

The published article includes all code generated or analyzed during this study. The data matrix used to generate the figures is attached as Extended data table 3, while the NRSA tool is hosted on GitHub (GitHub - vermeulenlab/lncRNA) due to discontinuation of hosting of the original authors.

## Supplemental information

Table S1. Buffers for nuclei isolation. Composition of buffers for nuclei isolation, related to step 1

Table S2. Buffers to perform run-on reactions. Composition of buffers for run-on reactions, related to step 9

Table S3. Buffers for bead preparation. Composition of buffers to prepare anti-BrdU beads, related to step 19

Data S1. Ordered csv.html: Python code to create an ordered and easily readable csv file of all the guides generated for a transcript combined with BLAT output, related to step 88

Data S2. Cleanup and guide picking.html: Python code to automatically pick a user-input amount of highest-scoring CRISPRi guides spread across a transcript, with an option to also add homology arms to the output, related to step 89

Data S3. Merge files.html: Python code to merge all the automatically picked transcripts into one file for ordering, related to step 89
