# Supplementary material for: Identifying essential long non-coding RNAs in cancer using CRISPRi-based dropout screens
Source: STAR Protoc. 2023 Sep 28;4(4):102588. doi: 10.1016/j.xpro.2023.102588 (PMC10550846; doi:10.1016/j.xpro.2023.102588)
Supplement: Data S1. Ordered csv.html: Python code to create an ordered and easily readable csv file of all the guides generated for a transcript combined with BLAT output, related to step 88 [file mmc4.zip › Ordered csv.html]

Ordered csv


In [1]:

```
import os
from os import listdir
from os.path import isfile, join
import pandas as pd
import numpy as np
import re
```

In [2]:

```
savepath = "./Clean/"
mypath = "./"
onlyfiles = [f for f in listdir(mypath) if isfile(join(mypath, f))]
onlycsv = [c for c in onlyfiles if ".csv" in c]
onlybed = [b for b in onlyfiles if ".bed" in b]
onlycsv = sorted(onlycsv)
onlybed = sorted(onlybed)
```

In [3]:

```
for csv, bed in zip(onlycsv, onlybed):
    data = pd.read_csv(mypath + csv, sep = "\t", header=None, names = ['a', 'b', 'c', 'd', 'e', 'f', 'g', 'h', 'i', 'j', 'k', 'l'], low_memory=False)
    scores = []
    for x in data['e']:
        if type(x) == str:
            if "0." in x:
                scores.append(x)
    guidenames = [None] * len(scores)
    for i in range(0,len(scores)):
        guidenames[i] = "T" + str(i+1)
    dfdict = {"guidenames" : guidenames,
             "guidescores" : scores}
    df = pd.DataFrame(dfdict)
    df2 = pd.read_csv(mypath + bed, sep='\t', comment='t', header=None)
    header = ['chrom', 'chromStart', 'chromEnd', 'name', 'score', 'strand']
    df2.columns = header[:len(df2.columns)]
    newstart = []
    newend = []
    smallest = min(df2["chromStart"])
    for coord in df2["chromStart"]:
        new_val = coord - smallest
        newstart.append(new_val)
    for coordi in df2["chromEnd"]:
        new_val2 = coordi-smallest
        newend.append(new_val2)
    mask = np.column_stack([data[col].str.contains(r"T\d", na=False) for col in data])
    result = data.loc[mask.any(axis=1)]
    sequences = []
    for seq in result['b']:
        sequences.append(seq)
    df3 = pd.DataFrame(newstart, columns = ["chromStart"])
    df4 = pd.DataFrame(newend, columns = ["chromEnd"])
    df5 = pd.DataFrame(sequences, columns = ["sequence"])
    df_clean = df.join(df3)
    df_clean = df_clean.join(df4)
    df_clean = df_clean.join(df5)
    df_sort = df_clean.sort_values(by="chromStart")
    df_sort.to_csv("./Clean/" + csv, index=False, header=True)
```

In [ ]:

```

```
