# Supplementary material for: Identifying essential long non-coding RNAs in cancer using CRISPRi-based dropout screens
Source: STAR Protoc. 2023 Sep 28;4(4):102588. doi: 10.1016/j.xpro.2023.102588 (PMC10550846; doi:10.1016/j.xpro.2023.102588)
Supplement: Data S2. Cleanup and guide picking.html: Python code to automatically pick a user-input amount of highest-scoring CRISPRi guides spread across a transcript, with an option to also add homology arms to the output, related to step 89 [file mmc5.zip › Cleanup and guide picking.html]

Cleanup and guide picking


In [3]:

```
import os
from os import listdir
from os.path import isfile, join
import pandas as pd
import numpy as np
import re
```

In [4]:

```
#Path of output files after using BLAT on the output fasta files

blatpath = "./fasta/Blat/"
blatfiles = [f for f in listdir(blatpath) if isfile(join(blatpath, f))]
onlypsl = [c for c in blatfiles if ".psl" in c]
nopsl = [q.split('.psl')[0] for q in onlypsl]
onlypsl = sorted(onlypsl)
nopsl = sorted(nopsl)
headers = ["query", "subject", "%id", "alignmentlength",
"mismatches", "gap openings", "query start", "query end", "subject start", "subject end", "E value", "bit score"]

#First path is the path where you want the cleaned up .csv files, second is the path of the .csv files output from
#Cas13design, third is the path of the counted BLAT hits files

mypath = "./Clean/"
outpath = "./Final/"
onlyfiles = [f for f in listdir(mypath) if isfile(join(mypath, f))]
onlycsv = [c for c in onlyfiles if ".csv" in c]
onlycsv = [q for q in onlycsv if "ENSG" in q]
onlycsv = sorted(onlycsv)
```

In [11]:

```
for psl, csv in zip(onlypsl, onlycsv):
    #This block counts the amount of blat hits of 22 nucleotides or more 
    datapsl = pd.read_csv(blatpath + psl, delimiter="\t", names = headers)
    data2 = pd.read_csv(mypath + csv)
    only22 = datapsl[lambda x: x['alignmentlength'] > 21]
    only22 = only22[lambda x: x['alignmentlength'] < 24]
    amount = only22['query'].value_counts()
    a = sorted(amount.index, key=lambda x: int("".join([i for i in x if i.isdigit()])))
    amount = amount.reindex(index=a)
    only22['blathits'] = ""
    for x, y in zip(amount.index, amount):
        b = only22.iloc[np.where(only22['query'] == x)].index
        for c in b:
            only22.at[c, 'blathits'] = y
    only22.sort_values(by = ['blathits', 'query'])
    only22.drop_duplicates(subset='query', inplace=True)
    only22 = only22.reset_index()
    only22.drop('index', inplace=True, axis=1)
    
    #This block cleans the .csv files and adds the amount of blathits in a new column

    full_csv = data2.join(only22['blathits'])
    data = full_csv.sort_values('chromStart')
    
    #This block automatically cuts the transcript into 10 equal-sized fragments, and picks the highest-scoring guide
    #with only 1 blat hit for each fragment. Also provides list of guides to order with an editable prepend and 
    #append sequence for homology arms.
    
    maxpos = int(max(data['chromStart']))
    minpos = int(min(data['chromStart']))
    length = maxpos - minpos
    fraction = round(length/10)
    brackets = []
    for i in range(0,11):
        brackets.append(i*fraction)
    brackets[0] = minpos
    brackets[10] = maxpos
    column_names = ["guidenames", "guidescores", "chromStart", "chromEnd", "sequence", "blathits"]
    frames = []
    for j in range(0,10):
        sliced = data.loc[data['chromStart'].between(brackets[j], brackets[j+1])]
        sortedd = sliced.sort_values(by='guidescores', ascending=False)
        for a in sortedd['guidescores']:
            looker = None
            looker = data.loc[data['guidescores'] == a]
            indexx = data.index[data['guidescores'] == a].tolist()
            ix = indexx[0]
            if len(looker) > 1 or "TTTT" in looker['sequence'][ix]:
                continue
            elif looker['blathits'].item() == 1:
                guide = data.loc[data['guidescores'] == a]
                break
            else:
                guide = pd.DataFrame(columns = column_names)
        frames.append(guide)
    
    df = pd.concat(frames)
    df = df.sort_values(by='chromStart')
    df['OrderSeq'] = df['sequence']
    df['TrimSeq'] = df['sequence']
    for x in data['sequence']:
        ind = data.loc[data['sequence'] == x].index[0]
        x = x[:-3]
        data.at[ind, 'TrimSeq'] = x
    
    #Edit these sequences to include homology arms for ordering
    
    preseq = "TATCTTGTGGAAAGGACGAAACACC"
    postseq = "GTTTTAGAGCTAGAAATAGCAAGTTAAAAT"
    df['OrderSeq'] = preseq + "G" + df['TrimSeq'].astype(str) + postseq
    
    #Export csv to the path specified
    df.to_csv(outpath + csv, index=False, header=True)
    #Resets all the values
    
    data = pd.DataFrame()
    df = pd.DataFrame()
    guide = pd.DataFrame()
```
