# Supplementary material for: Identifying essential long non-coding RNAs in cancer using CRISPRi-based dropout screens
Source: STAR Protoc. 2023 Sep 28;4(4):102588. doi: 10.1016/j.xpro.2023.102588 (PMC10550846; doi:10.1016/j.xpro.2023.102588)
Supplement: Data S3. Merge files.html: Python code to merge all the automatically picked transcripts into one file for ordering, related to step 89 [file mmc6.zip › Merge files.html]

Merge files


In [ ]:

```
import os
from os import listdir
from os.path import isfile, join
import pandas as pd
import numpy as np
import re
```

In [ ]:

```
outpath = "./Final/"
onlyfiles = [f for f in listdir(outpath) if isfile(join(outpath, f))]
onlycsv = [c for c in onlyfiles if ".csv" in c]
onlycsv = [q for q in onlycsv if "ENSG" in q]
onlycsv = sorted(onlycsv)
```

In [ ]:

```
##Merge all the cleaned up guide csvs into a single .csv file

with open(outpath + "Merged.csv", "w+") as f:
    for csv in onlycsv:
        data = pd.read_csv(outpath + csv, sep = ",", header =None)
        data.columns = [re.sub('\.csv$', '', csv), '', '' , '' , '' , '', '']
        data.to_csv(f, index = False, header=True)
        f.write("\n" *2)
```
